# Supplementary material for: γ-TuRC Heterogeneity Revealed by Analysis of Mozart1
Source: Curr Biol. 2018 Jul 23;28(14):2314–2323.e6. doi: 10.1016/j.cub.2018.05.044 (PMC6065531; doi:10.1016/j.cub.2018.05.044)
Supplement: Document S1. Figures S1–S4 and Table S1 [file mmc1.pdf]

Current Biology, Volume *28*

## Supplemental Information

# $\gamma$-TuRC Heterogeneity Revealed by Analysis of Mozart1

**Corinne A. Tovey, Chloe E. Tubman, Eva Hamrud, Zihan Zhu, Anna E. Dyas, Andrew N. Butterfield, Alex Fyfe, Errin Johnson, and Paul T. Conduit**

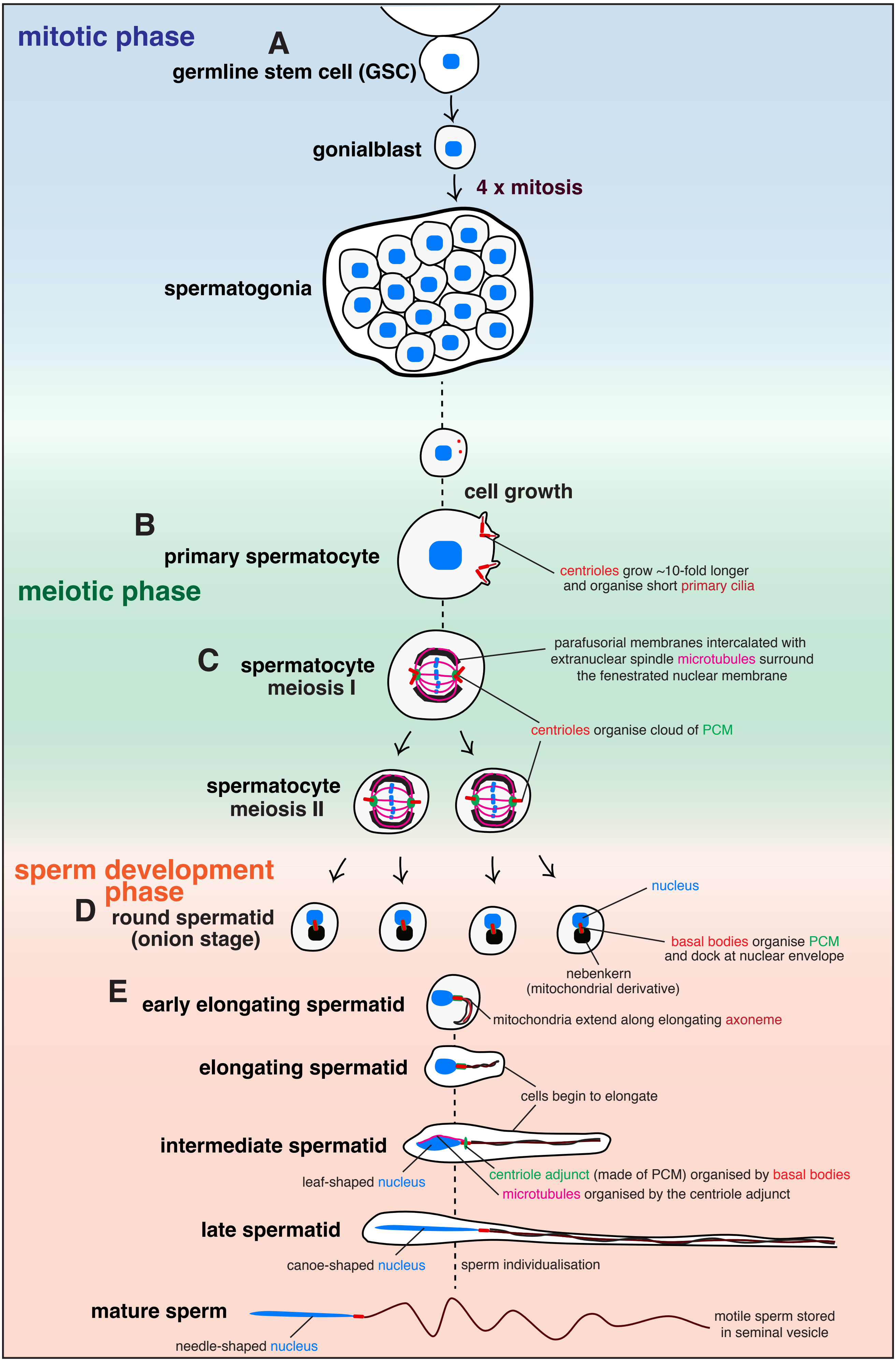
mitotic phase
A
germline stem cell (GSC)
gonialblast
4 x mitosis
spermatogonia
cell growth
B
primary spermatocyte
meiotic phase
centrioles grow ~10-fold longer and organise short primary cilia
C
spermatocyte meiosis I
parafusorial membranes intercalated with extranuclear spindle microtubules surround the fenestrated nuclear membrane
centrioles organise cloud of PCM
spermatocyte meiosis II
sperm development phase
D
round spermatid (onion stage)
nucleus
basal bodies organise PCM and dock at nuclear envelope
nebenkern (mitochondrial derivative)
E
early elongating spermatid
mitochondria extend along elongating axoneme
elongating spermatid
cells begin to elongate
intermediate spermatid
leaf-shaped nucleus
centriole adjunct (made of PCM) organised by basal bodies
microtubules organised by the centriole adjunct
late spermatid
canoe-shaped nucleus
sperm individualisation
mature sperm
needle-shaped nucleus
motile sperm stored in seminal vesicle

**Figure S1. Stages of spermatogenesis in *Drosophila*, related to Figures 2 and 3.** Cartoon summarises the various stages of spermatogenesis in *Drosophila*. (**A**) Spermatogenesis starts when a germline stem cell divides to produce a gonialblast. Gonialblasts go through four rounds of synchronous mitosis to produce cysts of 16 interconnected spermatogonia. (**B**) Each cell matures and grows in size to become a primary spermatocyte. The centrioles (red) increase ~10-fold in length and become resolvable by standard-resolution light microscopy. They are positioned at the cell cortex during interphase and nucleate short primary cilia. (**C**) As the cells enter meiosis the centrioles migrate to the nuclear envelope and recruit large amounts of PCM, which contains γ-TuRCs that nucleate large numbers of cytoplasmic microtubules. The nuclear envelope partially breaks down close to the centrosomes and spindle microtubules penetrate the nucleus; most spindle microtubules, however, remain outside the nuclear envelope and intercalate with a series of double membranes called the parafusorial membranes. (**D**) After the second meiotic division, the centrioles, soon to become basal bodies, are inherited by the round spermatid cells and become anchored at the nuclear envelope, positioned between the nucleus and mitochondrial derivative (nebenkern) in a Dynein-dependent manner. The spermatids remain as 64-cell cysts until sperm individualisation at the end of spermatogenesis. (**E**) In early elongating spermatids, the two mitochondrial derivatives from the nebenkern extend and wrap around the axoneme, which grows from the basal body. The cells begin to elongate and in intermediate spermatids the PCM is reorganised into the centriole adjunct. The basal body embeds into an indentation of the nuclear envelope called the nuclear socket at the basal tip of the nucleus, and cytoplasmic microtubules emanate from the centriole adjunct and run along the outer nuclear membrane to form part of the dense complex. Microtubules are also nucleated by γ-TuRCs that are recruited to the surface of the mitochondrial derivatives. In late spermatids the centriole adjunct dissipates, the nuclei progressively become needle-like in shape, and the cells are eventually separated into individual sperm; they become motile and are stored in the seminal vesicles. The mitochondrial derivatives persist along the length of the sperm tails throughout sperm differentiation.

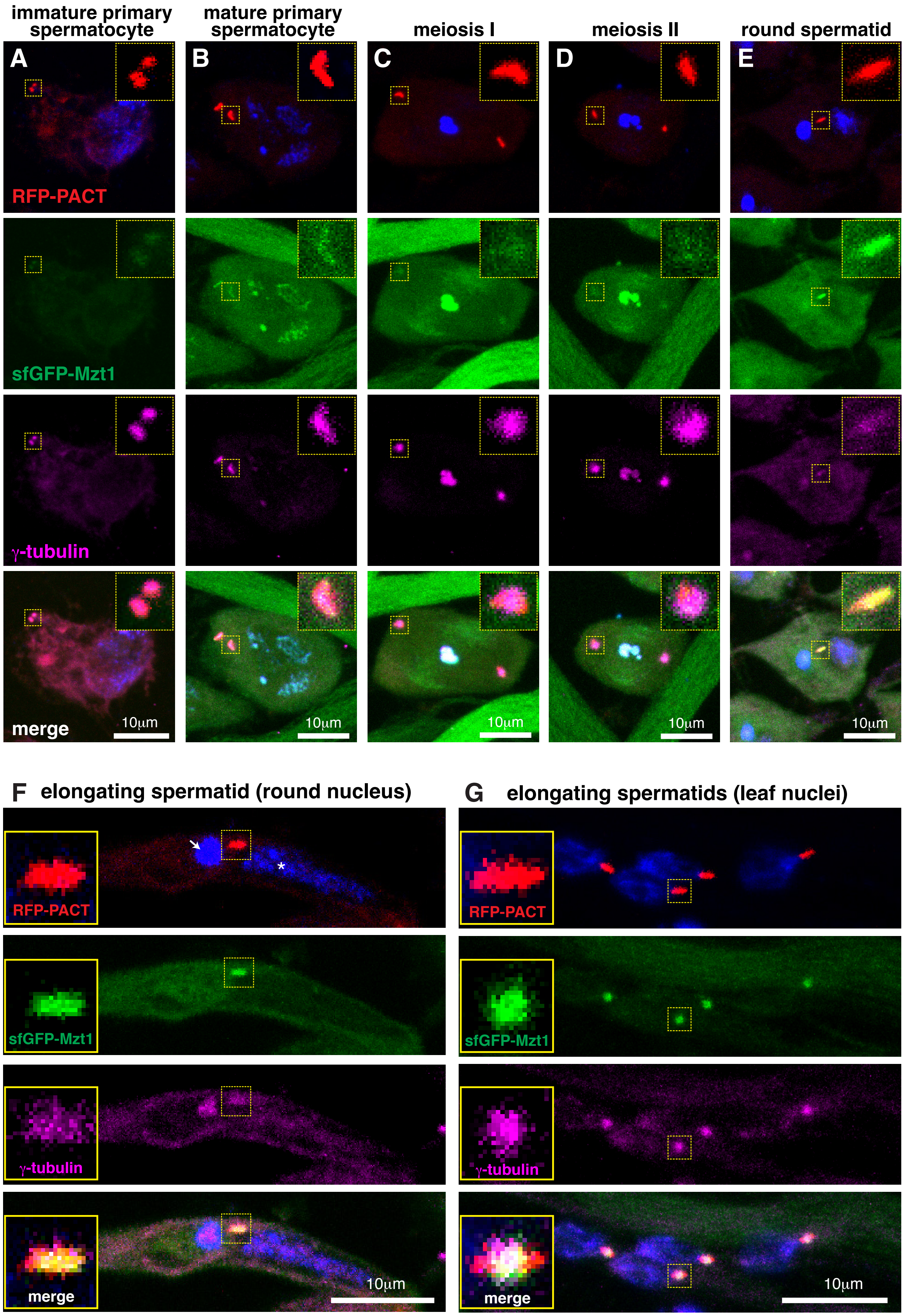
immature primary spermatocyte
mature primary spermatocyte
meiosis I
meiosis II
round spermatid
A
B
C
D
E
RFP-PACT
sfGFP-Mzt1
γ-tubulin
merge
10μm
F elongating spermatid (round nucleus)
G elongating spermatids (leaf nuclei)
RFP-PACT
sfGFP-Mzt1
γ-tubulin
merge
10μm

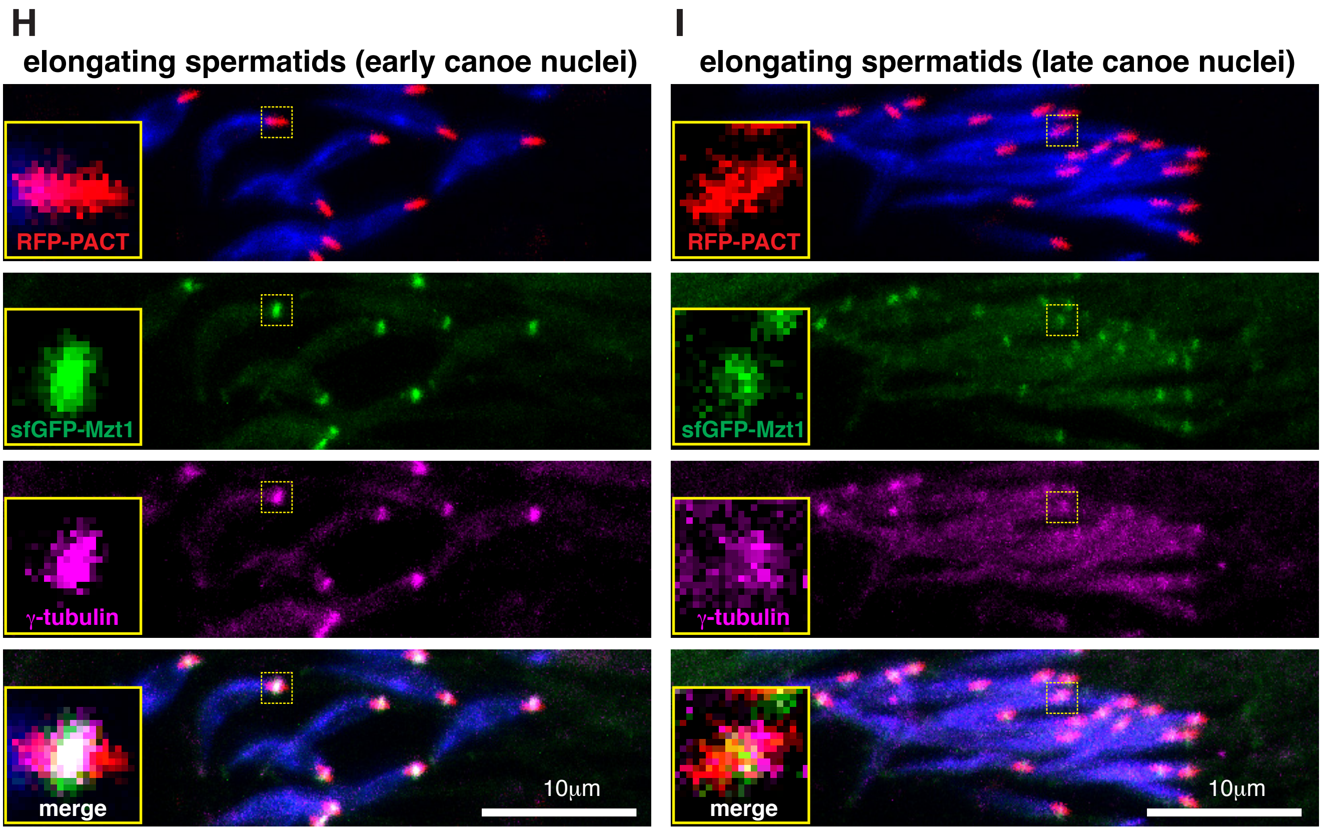
H
elongating spermatids (early canoe nuclei)
RFP-PACT
sfGFP-Mzt1
γ-tubulin
merge
10μm
I
elongating spermatids (late canoe nuclei)
RFP-PACT
sfGFP-Mzt1
γ-tubulin
merge
10μm

**Figure S2. Analysis of γ-tubulin and sfGFP-Mzt1 localisation during spermatogenesis from spermatocytes to round spermatids, related to Figure 2.** Testes expressing sfGFP-Mzt1 (green) and RFP-PACT (red) were fixed and stained for γ-tubulin (magenta) and DNA (blue). RFP-PACT marks centrioles and basal bodies. Images of cells at different developmental stages (as indicated at the top of each panel) are displayed in temporal order (earliest stage on the left); note that images of spermatogonial cells, where sfGFP-Mzt1 is not detected, are not shown due to space constraints. The centrosomes (A-D) or basal bodies (E-I) are enlarged in the yellow boxes in each image. (**A**) sfGFP-Mzt1 is present at very low levels in immature spermatocytes but does co-localise with γ-tubulin at centrioles. (**B**-**D**) sfGFP-Mzt1 is present at higher levels in mature spermatocytes and co-localises with γ-tubulin at the centrioles. Note that whereas the γ-tubulin is strongly concentrated around the centrioles in meiotic spermatocytes, the sfGFP-Mzt1 signal is relatively weak (**C**,**D**). Note also that the bright sfGFP-Mzt1 signal at the chromatin in (C) and (D) is likely due to "bleed through" from the Hoechst signal, as we do not observe it in live specimens (see Figure 2G,H). (**E**) sfGFP-Mzt1 concentrates strongly at basal bodies in round spermatids, co-localising with a relatively weak signal of γ-tubulin. (**F**) Images show a very early elongating spermatid where the nucleus (arrow) is still round but the mitochondrial derivatives (asterisk) have started to elongate along the sperm tail. sfGFP-Mzt1 and γ-tubulin localise along the length of the basal body. (**G**) Images show a group of early spermatids, as judged by the leaf-shape nuclei. sfGFP-Mzt1 and γ-tubulin localise at the centriole adjunct, which forms a ring around the centre of the basal body. (**H**) Images show a group of intermediate spermatids where the nuclei have become canoe-shaped. sfGFP-Mzt1 and γ-tubulin still colocalise at the centriole adjunct. (**I**) Images show a group of more mature spermatids where the nuclei have elongated further (late canoe nuclei) and both sfGFP-Mzt1 and γ-tubulin have started to dissipate from the basal body.

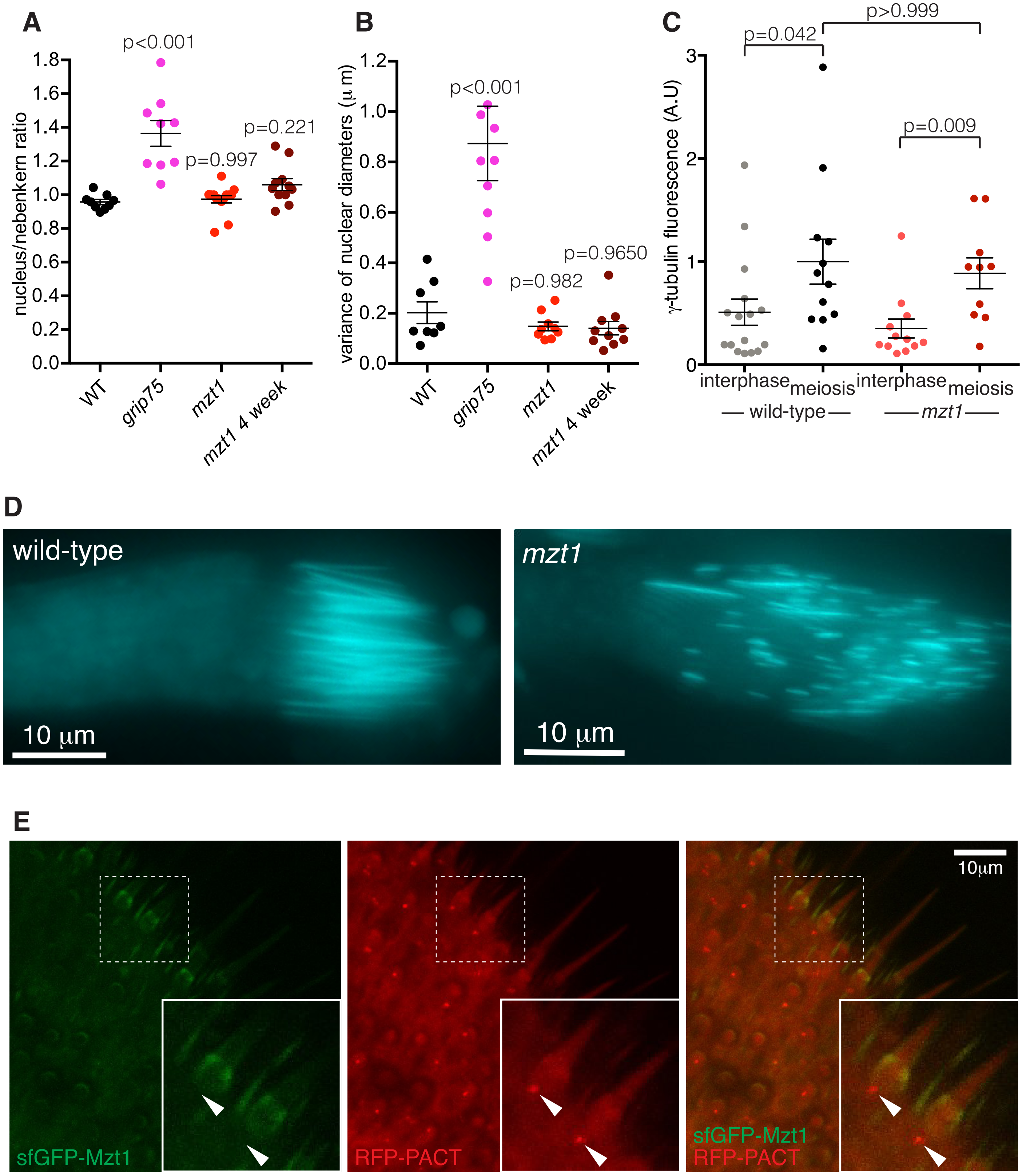

A
p<0.001
p=0.997
p=0.221
nucleus/nebenkern ratio
1.8
1.6
1.4
1.2
1.0
0.8
0.6
0.4
0.2
0.0
WT
grip75
mzt1
mzt1 4 week
B
p<0.001
p=0.982
p=0.9650
variance of nuclear diameters (μ m)
1.2
1.0
0.8
0.6
0.4
0.2
0.0
WT
grip75
mzt1
mzt1 4 week
C
p=0.042
p>0.999
p=0.009
γ-tubulin fluorescence (A.U)
3
2
1
0
interphase
meiosis
interphase
meiosis
wild-type
mzt1
D
wild-type
10 μm
mzt1
10 μm
E
10μm
sfGFP-Mzt1
RFP-PACT
sfGFP-Mzt1
RFP-PACT

**Figure S3. Mzt1 is required for proper nuclei positioning in spermatids, but not for male meiosis, and does not localise to the basal bodies of cilia in sensory neurons, related to Figure 3.** (**A**,**B**) Graphs show the results of an analysis of onion stage round spermatid cysts from pupal wild-type, pupal *grip75* mutant, pupal *mzt1* mutant or 4-week-old *mzt1* mutant flies, as indicated. Each data-point represents an average value from a cyst of cells. *mzt1* mutant cysts have a ~1:1 nucleus:nebenkern ratio (A) and display little variation in nuclear size (B), indicating that meiosis proceeds normally. (**C**) Graph shows the quantification of centrosomal γ-tubulin fluorescence in interphase and meiotic wild-type and *mzt1* mutants, as indicated; each data-point represents an average value from a cyst of cells. Note that the centrosomes in *mzt1* mutant meiotic spermatocytes can still recruit large amounts of γ-tubulin. (**D**) Images show spermatid bundles from live squashes of either wild-type (left) or *mzt1* mutant (right) testes incubated with Hoechst (DNA, blue). Autofluorescence reveals the position of the sperm tails. While the nuclei are all positioned at the tip of the bundle in the wild-type, they are more scattered in the *mzt1* mutant. Note that the Hoechst staining is not always evenly distributed along the length of an individual nucleus, such that the two ends can be brighter than the middle. (**E**) Antennae from flies co-expressing sfGFP-Mzt1 and RFP-PACT (a basal body marker) were dissected, fixed and mounted; images of the edge of the third antennal segment were taken on a confocal microscope. The insets in the bottom right of each image are enlargements of the boxed regions and the arrowheads point to basal bodies. Error bars represent SEM.

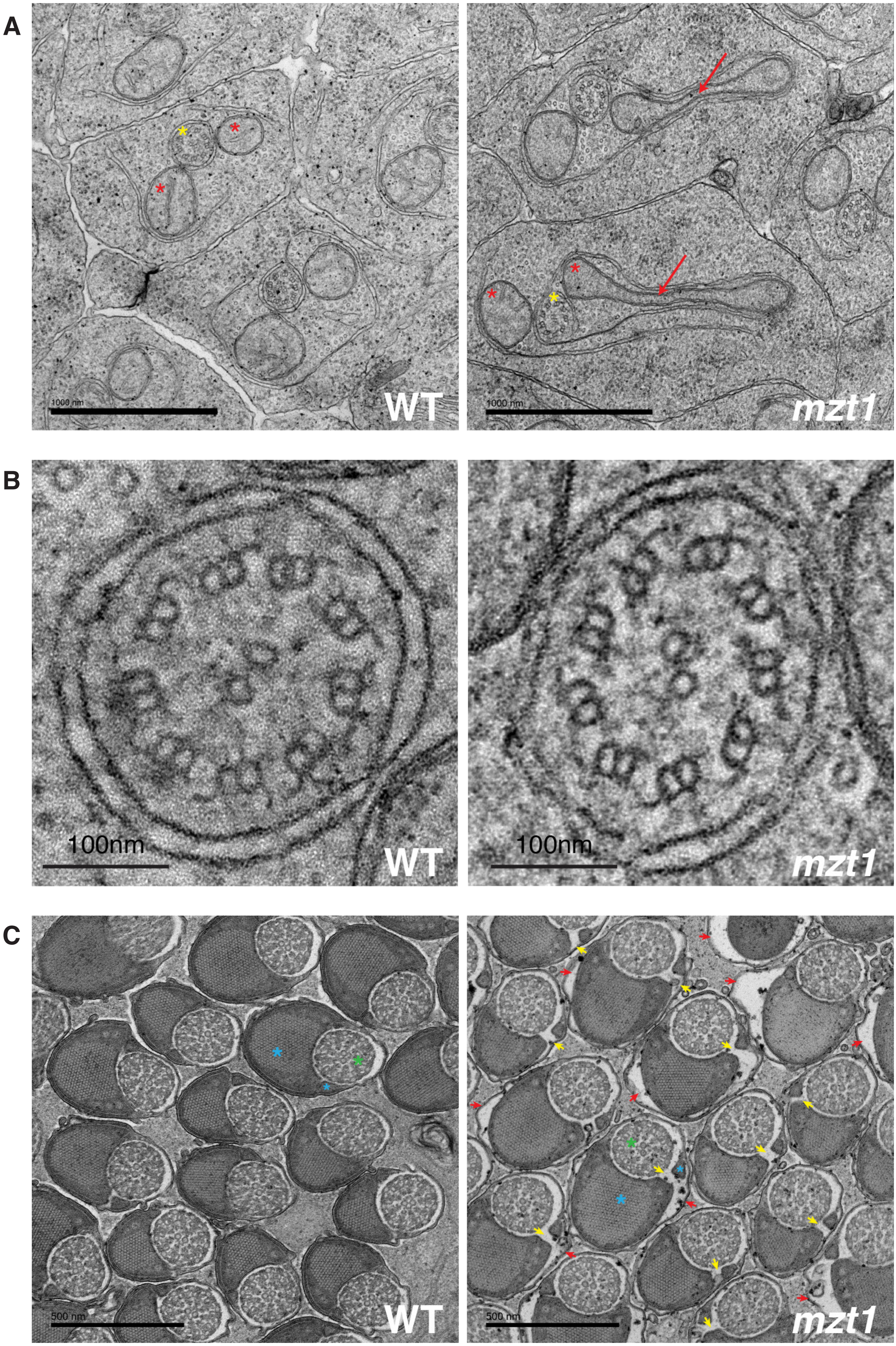
A
1000 nm
WT
1000 nm
mzt1
B
100nm
WT
100nm
mzt1
C
500 nm
WT
500 nm
mzt1

**Figure S4. Transmission electron microscopy imaging of sperm tails reveals ultrastructural defects in *mzt1* mutants, related to Figure 3.** (**A**) Images show cross sections of sperm tails from intermediate spermatids in either wild-type (left) or *mzt1* mutant (right) pupal testes. Axonemes and mitochondrial derivatives from an individual tail in each case are marked with a yellow asterisk and red asterisks, respectively. Misshapen, severely elongated mitochondrial derivatives in the mutant are indicated with red arrows. This type of severe elongation was seen in 10 out of 36 sperm tails examined in the mutant, but in only 1 out of 43 sperm tails in the wild-type. (**B**) Images show cross sections of axonemes from intermediate spermatids in either wild-type (left) or *mzt1* mutant (right) pupal testes. A 9+2 arrangement of microtubules can be seen in both cases. (**C**) Images show cross sections of individualised sperm tails from mature spermatids in either wild-type (left) or *mzt1* mutant (right) testes from 4-week-old flies. Axonemes and the major and minor mitochondrial derivatives from one of the tails in each case are marked with a green asterisk and blue asterisks, respectively. In the mutant, red arrowheads indicate where the membrane has separated from the major mitochondrial derivative (this occurred in 84.9% mutant tails, n=212, and in only 14.7% wild-type tails, n=68). Yellow arrowheads indicate unusually large gaps between the major and minor mitochondrial derivatives (this occurred in 71.7% mutant tails, n=212, and in only 4.4% wild-type tails, n=68).

| Drosophila | Human |
| --- | --- |
| $\gamma$-tubulin37C (maternal)<br>$\gamma$-tubulin23C (ubiquitous) | $\gamma$-tubulin1 and $\gamma$-tubulin2 |
| Grip84 | GCP2 |
| Grip91 | GCP3 |
| Grip75 | GCP4 |
| Grip128 | GCP5 |
| Grip163 | GCP6 |
| Grip71 | NEDD1 / GCP-WD / GCP7 |
| - | MOZART2A / GCP8A |
| - | MOZART2B / GCP8B |
| CG42787 | MOZART1 / GCP9 |
| nmdyn-D7 | NME7 / NDK7 |
| ? | LGALS3BP |

**Table S1. Homologues of γ-TuRC proteins in *Drosophila* and human cells, related to Figures 1 and 3.**
