# Supplementary material for: γ-TuRC Heterogeneity Revealed by Analysis of Mozart1
Source: Curr Biol. 2018 Jul 23;28(14):2314–2323.e6. doi: 10.1016/j.cub.2018.05.044 (PMC6065531; doi:10.1016/j.cub.2018.05.044)
Supplement: Document S2. Article plus Supplemental Information [file mmc5.pdf]

Report

Current Biology

γ-TuRC Heterogeneity Revealed by Analysis of Mozart1

Graphical Abstract

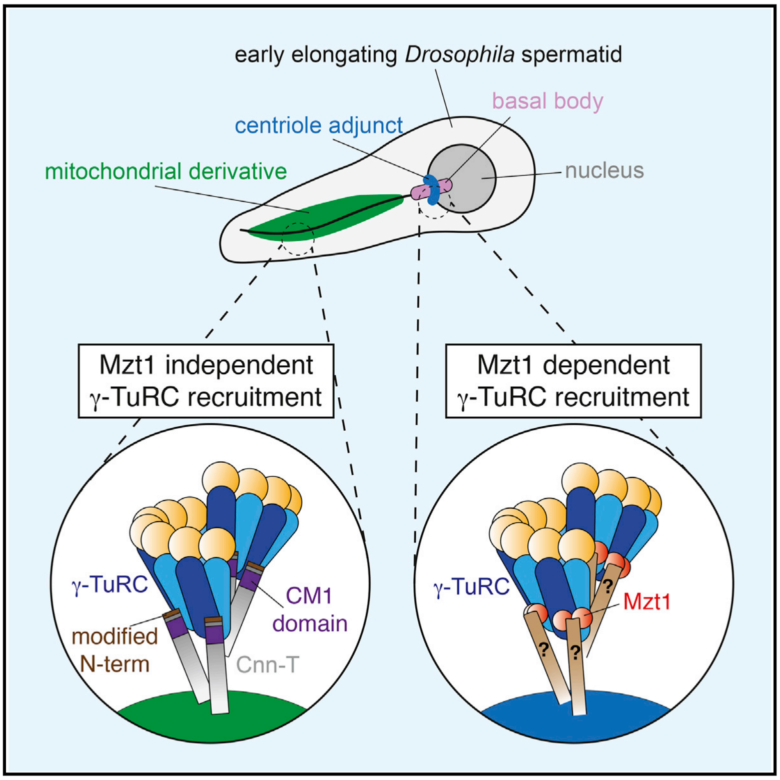

Highlights

- The protein composition of gamma-tubulin ring complexes (γ-TuRCs) varies in flies
- *Drosophila* Mozart1 is non-essential, and its expression is restricted to the testes
- Mozart1 is required for γ-TuRC recruitment specifically to basal bodies
- *mzt1* mutants have defects in basal body positioning and sperm formation

Authors

Corinne A. Tovey, Chloe E. Tubman, Eva Hamrud, ..., Alex Fyfe, Errin Johnson, Paul T. Conduit

Correspondence

ptc29@cam.ac.uk

In Brief

Tovey et al. show that γ-TuRCs do not all have the same protein composition in *Drosophila*. This heterogeneity is determined by the γ-TuRC protein Mzt1, which is expressed only in fly testes. Mzt1 is surprisingly not required for cell division but is instead required for the recruitment of γ-TuRCs to basal bodies and for correct sperm formation.

Tovey et al., 2018, Current Biology *28*, 2314–2323
July 23, 2018 © 2018 The Author(s). Published by Elsevier Ltd.
https://doi.org/10.1016/j.cub.2018.05.044

CellPress

Current Biology
Report

γ-TuRC Heterogeneity Revealed by Analysis of Mozart1

Corinne A. Tovey,[1] Chloe E. Tubman,[1] Eva Hamrud,[1] Zihan Zhu,[1] Anna E. Dyas,[1] Andrew N. Butterfield,[1] Alex Fyfe,[2] Errin Johnson,[2] and Paul T. Conduit[1,3,*]
[1]Department of Zoology, University of Cambridge, Downing Street, Cambridge CB2 3EJ, UK
[2]Sir William Dunn School of Pathology, University of Oxford, South Parks Road, Oxford OX1 3RE, UK
[3]Lead Contact
*Correspondence: ptc29@cam.ac.uk
https://doi.org/10.1016/j.cub.2018.05.044

SUMMARY

Microtubules are essential for various cell processes [1] and are nucleated by multi-protein γ-tubulin ring complexes (γ-TuRCs) at various microtubule organizing centers (MTOCs), including centrosomes [2–6]. Recruitment of γ-TuRCs to different MTOCs at different times influences microtubule array formation, but how this is regulated remains an open question. It also remains unclear whether all γ-TuRCs within the same organism have the same composition and how any potential heterogeneity might influence γ-TuRC recruitment. MOZART1 (Mzt1) was recently identified as a γ-TuRC component [7, 8] and is conserved in nearly all eukaryotes [6, 9]. Mzt1 has so far been studied in cultured human cells, yeast, and plants; its absence leads to failures in γ-TuRC recruitment and cell division, resulting in cell death [7, 9–15]. Mzt1 is small (~8.5 kDa), binds directly to core γ-TuRC components [9, 10, 14, 15], and appears to mediate the interaction between γ-TuRCs and proteins that tether γ-TuRCs to MTOCs [9, 15]. Here, we use *Drosophila* to investigate the function of Mzt1 in a multicellular animal for the first time. Surprisingly, we find that *Drosophila* Mzt1 is expressed only in the testes and is present in γ-TuRCs recruited to basal bodies, but not to mitochondria, in developing sperm cells. *mzt1* mutants are viable but have defects in basal body positioning and γ-TuRC recruitment to centriole adjuncts; sperm formation is affected and mutants display a rapid age-dependent decline in sperm motility and male fertility. Our results reveal that tissue-specific and MTOC-specific γ-TuRC heterogeneity exist in *Drosophila* and highlight the complexity of γ-TuRC recruitment in a multicellular animal.

RESULTS AND DISCUSSION

Drosophila contains homologs of nearly all known γ-TuRC components (Table S1) [6]. The only predicted *D. melanogaster* homolog of Mzt1 is CG42787 [10, 12, 14], which we confirmed with extensive BLAST searches. CG42787 is 82 amino acids long, and its central region (D^{12} to R^{57}) is most similar to Mzt1 homologs in other species (Figure 1A).

To test whether CG42787 (hereafter Mzt1) can associate with the γ-TuRC in *Drosophila*, we generated transgenic flies expressing sfGFP-Mzt1 under the control of the polyubiquitin promoter (pUbq-sfGFP-Mzt1). pUbq-sfGFP-Mzt1 localized to centrosomes and spindles in syncytial embryos (Video S1), and γ-tubulin and Grip71, two known γ-TuRC proteins, co-immunoprecipitated with sfGFP-Mzt1 from embryo extracts (Figure 1B). sfGFP-Mzt1 also co-sedimented with γ-tubulin in both mid-density (γ-TuSC) and high-density (γ-TuRC) fractions during sucrose gradient sedimentation experiments (Figure 1C). To test whether *Drosophila* Mzt1 interacts with the same part of the γ-TuRC as Mzt1 homologs in other species, we performed a yeast-two-hybrid (Y2H) analysis. Y2H experiments in human cells have shown that Mzt1 strongly interacts with the N-terminal extension (NTE) regions of GCP3, GCP5, and GCP6 that precede the first Grip domain in each protein [9, 15]. We found that, although *Drosophila* Mzt1 interacted only weakly with the NTE region of Grip91 (GCP3) and failed to interact with the NTE region of Grip163 (GCP6), it interacted strongly with the NTE region of Grip128 (GCP5; Figure 1D). We conclude that CG42787 is the *D. melanogaster* homolog of Mzt1 and that it binds to the γ-TuRC in a similar, but potentially not identical, way to human Mzt1.

In order to study Mzt1 function in flies, we generated two mutant alleles (*mzt1*^{11} and *mzt1*^{57}) by using CRISPR to delete the majority of the *mzt1* coding sequence. Importantly, both alleles lack the central conserved residues, and we therefore consider them null alleles (Figure 1A). Surprisingly, we found that *mzt1* mutants were viable and that adult flies displayed no obvious morphological defects (data not shown). Previous genome-wide mRNA expression data suggested, however, that *mzt1* expression is predominantly restricted to the testes in *Drosophila* [16, 17]. To test this at the protein level, we generated endogenously tagged sfGFP-Mzt1 lines and examined expression in different tissues. Consistent with the mRNA expression data, we found that sfGFP-Mzt1 was present in extracts from pupal and adult testes and at low levels in larval gonads but was absent from the other tissues tested, including those with high mitotic activity (Figure 1E). We therefore tested the fertility of *mzt1* mutant males and, to our surprise, found that young *mzt1* mutant males were only slightly less fertile

 © 2018 The Author(s). Published by Elsevier Ltd.
This is an open access article under the CC BY license (http://creativecommons.org/licenses/by/4.0/).

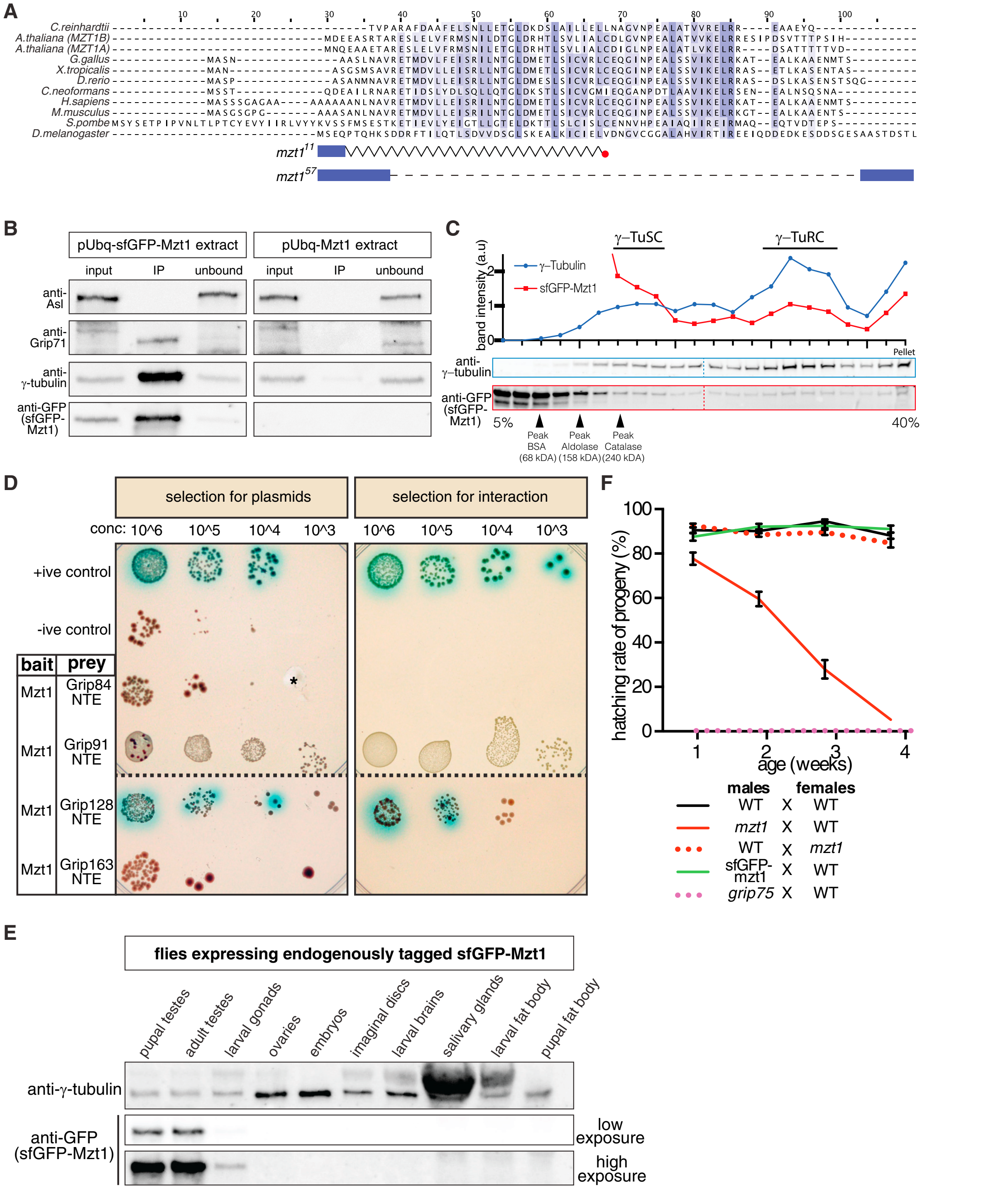

Figure 1. *Drosophila* Mzt1 Is a γ-TuRC Component Expressed Only in the Testes and Is Required for Proper Male Fertility

(A) A JalView multiple-protein alignment of Mzt1 homologs from different species (as indicated). Darker shading indicates higher similarity. The central region of *Drosophila* Mzt1, spanning amino acids D^{12} to R^{57}, is 29% sequence identical to human Mzt1. The diagram below indicates the protein sequence of the *mzt1[11]* and *mzt1[57]* mutant alleles generated in this study (blue boxes, presence of normal amino acids; zig-zag line, scrambled sequence induced by a frameshift; red dot, new stop codon; dashed line, deleted sequence).

(B) Western blots (probed with various antibodies as indicated) show results of anti-GFP immunoprecipitation from extracts of embryos expressing either pUbq-sfGFP-Mzt1 (left panels) or pUbq-Mzt1 (right panels). When sfGFP-Mzt1 is present in the extract, the anti-GFP antibodies co-immunoprecipitate sfGFP-Mzt1, γ-tubulin, and Grip71, but not Asl.

(C) Western blots and graph show results of fractionating extracts from embryos expressing pUbq-sfGFP-Mzt1 by sucrose gradient sedimentation. Gradient fractions were immunoblotted for γ-tubulin and GFP, and the position at which various markers ran on a parallel gradient are indicated. The graph plots the band

(legend continued on next page)

than wild-type males, in contrast to mutants for *grip75* (GCP4) (another non-essential γ-TuRC gene) (Figure 1F). However, the fertility of *mzt1* mutant males decreased dramatically with age, and they were sterile at ∼4 weeks old (Figure 1F). The fertility of wild-type males and males with sfGFP inserted at the endogenous *mzt1* locus remained high over this 4-week period (Figure 1F), showing that the sfGFP tag did not affect Mzt1 function. Moreover, the fertility of *mzt1* mutant females was also retained with age (Figure 1F), consistent with the lack of *mzt1* expression in the female germline. We conclude that *Drosophila* Mzt1 is expressed only in the testes and larval gonads and is required to maintain male fertility during aging.

We next wanted to examine the subcellular localization of Mzt1 in the testes. Spermatogenesis within *Drosophila* testes is characterized by a series of cell divisions and maturation stages [18] (Figure S1). We noticed that, typical of a testes-specific gene [19], the intensity of the sfGFP-Mzt1 signal increased with progression through spermatogenesis. There was no detectable signal in the apical tips of the testes (which contain the stem cells and mitotically dividing spermatogonia) (Figure 2A) and no signal at centrosomes in spermatogonia (data not shown). There was, however, a weak signal at centrosomes in spermatocytes (Figures 2B, 2D, 2G, 2H, and S2A–S2D) and a strong signal at basal bodies in spermatids that increased as spermatids aged (Figures 2C–2E and S2E–S2I). The pattern of sfGFP-Mzt1 at centrioles and basal bodies was similar to that reported for γ-tubulin (Figure 2D) [20–22], and immunostaining showed that sfGFP-Mzt1 colocalized with γ-tubulin in spermatocytes and spermatids at different developmental stages (Figures 2E and S2). The sfGFP-Mzt1 signal was particularly strong at centriole adjuncts that encircle the basal bodies in intermediate spermatids (Figures 2D, 2E, S2G, and S2H) and then spread along sperm tails proximal to the nuclei when the centriole adjuncts dissipated in later stage spermatids (Figure 2E, asterisk). Co-expression of Tag-RFP-Mzt1 and γ-tubulin-sfGFP showed that both exhibited a punctate pattern in sperm tails and these puncta often colocalized (Figure 2F). sfGFP-Mzt1 also localized to the few spindle microtubules that penetrate the nucleus in meiotic spermatocytes (Video S2) and to the extra-nuclear spindle microtubules that intercalate with the parafusorial membranes (Figures 2G and 2H; Video S2). Overall, these data show that Mzt1 colocalizes with γ-tubulin at centrioles and basal bodies throughout the majority of spermatogenesis, consistent with it being a member of the γ-TuRC in *Drosophila* testes.

We then examined *mzt1* mutants for any cellular defects that could explain their reduced fertility. Cysts of primary spermatocytes in *mzt1* mutants always contained 16 cells (8/8 cysts from 3 flies), indicating no defects in spermatogonial mitosis. We also found no evidence for defects in spermatocyte meiosis, as round spermatids (the products of male meiosis) nearly always contained a single nebenkern and a single nucleus of roughly equal size, even in testes from 4-week-old *mzt1* mutants (Figures 3A, S3A, and S3B). This is in contrast to mutations in most other γ-TuRC genes, including *grip75* (Figures 3A, S3A, and S3B) [23–26]. Consistent with the absence of nuclear size and number defects in round spermatids, centrosomes in *mzt1* mutant spermatocytes could organize robust microtubule asters during meiosis (Figure 3B) and could recruit wild-type levels of γ-tubulin during both interphase (Figure S3C) and meiosis (Figures 3C and S3C). Thus, Mzt1 is not required for the mitotic or meiotic divisions in the male germline.

After meiosis, dynein motors located at the nuclear envelope and nebenkern (a mitochondrial derivative) of round spermatids are predicted to pull on microtubules organized by the centriole (now a basal body), positioning it between the nucleus and nebenkern [27–30] (Figure 3D, arrow). We found that, compared with basal bodies in wild-type round spermatids, basal bodies in *mzt1* mutant round spermatids were more frequently mispositioned (Figures 3D and 3E) and recruited less γ-tubulin (Figures 3F and 3G). These defects were even more severe in elongating spermatids, where the basal bodies in *mzt1* mutant cells recruited much less γ-tubulin to the centriole adjuncts than in wild-type cells and were frequently mispositioned away from the basal tips of the elongating nuclei (Figures 3H–3J). Co-staining with antibodies against Mzt1 confirmed that Mzt1 was absent from the centriole adjunct in *mzt1* mutants (Figure 3I). Intriguingly, these basal body positioning defects increased with fly age (Figure 3K), providing a potential explanation for the age-related decrease in male fertility (Figure 1F). In later stage spermatids, the needle-like nuclei were also often dispersed along the bundle in *mzt1* mutants (Figure S3D), similar to *grip75* and *grip128* mutants [23]. We conclude that defects in γ-tubulin recruitment to basal bodies in *mzt1* mutant spermatids are associated with, and may lead to, basal body positioning defects. Mzt1 does not, however, appear to have a more general role at basal bodies in flies, as sfGFP-Mzt1 did not localize to basal bodies of cilia in sensory neurons in the antennae (Figure S3E) and *mzt1* mutant adults of various ages displayed no obvious coordination defects (data not shown), which are normally associated with ciliary defects.

We next tested whether the age-dependent decrease in the fertility of male *mzt1* mutants was related to defects in sperm motility. As expected, young fertile *mzt1* mutant males contained

intensity of γ-tubulin (blue) and sfGFP-Mzt1 (red) relative to their median band intensity throughout the gradient. Note that, although sfGFP-Mzt1 can be found in the mid- and high-density fractions, there are also high levels of sfGFP-Mzt1 in the low density (cytosolic) fractions, probably due to high protein expression induced by the pUbq promoter; the stronger peak of γ-tubulin compared to sfGFP-Mzt1 in the high-density fractions may indicate that the γ-TuRCs in these embryos contain more molecules of γ-tubulin than sfGFP-Mzt1.

(D) Images show results of a yeast-two-hybrid analysis between Mzt1 (bait) and the N-terminal extension regions of different Grip proteins (prey). Mated yeast were plated as 10-fold serial dilutions (left to right) on DDO medium that selected for the bait and prey plasmids (left panels) or QDO medium that selected for both the plasmids and an interaction (right panels) (see STAR Methods). Yeast containing plasmids that do not interact produce a toxic red pigment, AIR1, and so grow poorly even on DDO medium. X-α-Gal, which generates a blue color when interactions occur, was included in both plate types. The asterisk indicates a gouge in the medium.

(E) Western blot of protein extracts from different *Drosophila* tissues (as indicated) probed with antibodies against γ-tubulin and GFP.

(F) Graph shows the results of fertility tests performed on male or female wild-type or mutant flies, as indicated. Error bars represent SEM.

See also STAR Methods, Table S1, and Video S1.

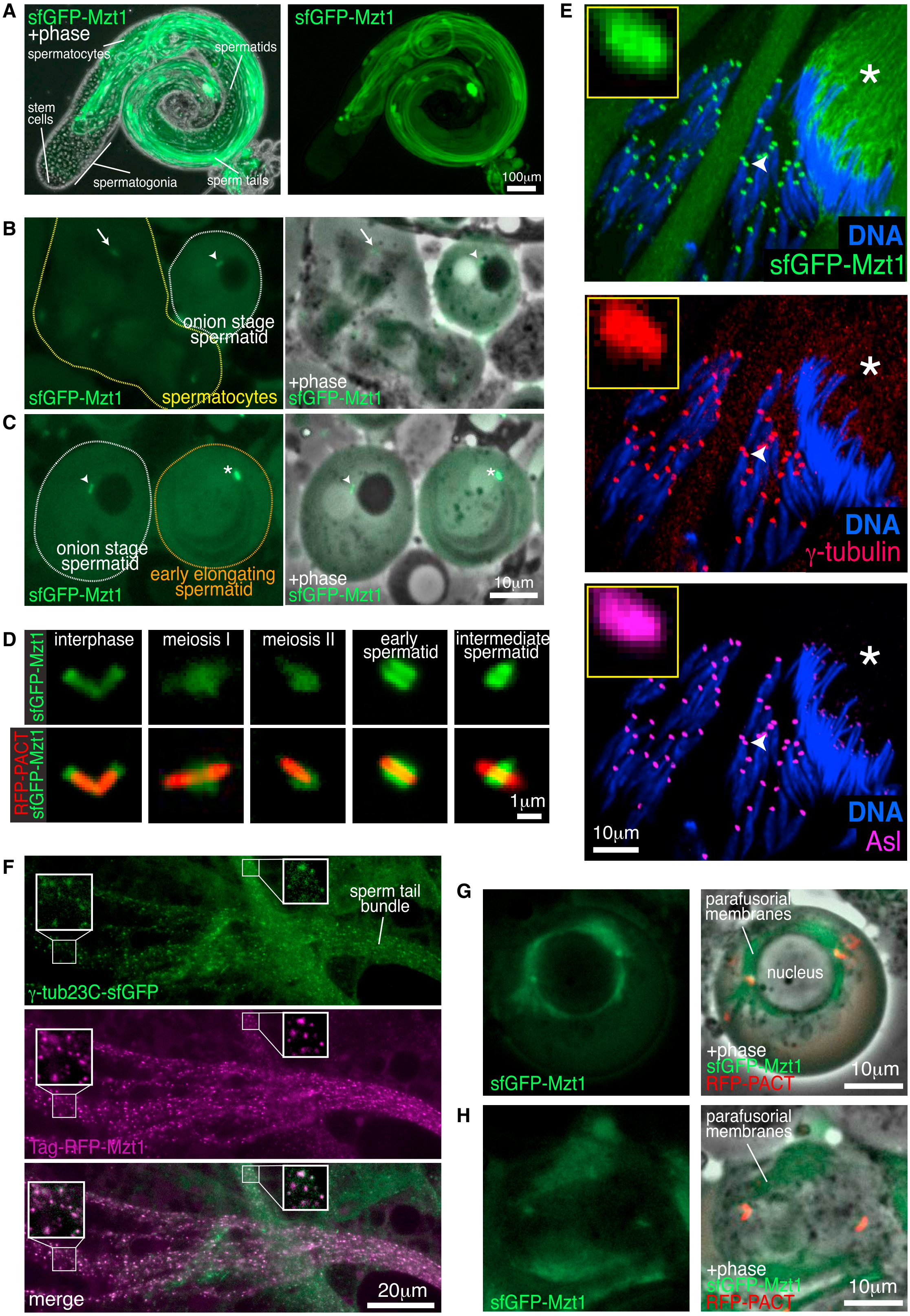

(legend on next page)

motile sperm in their seminal vesicles (n = 9/10; Video S3D), and females that had mated with these males contained large numbers of motile sperm in their sperm-storage organs (n = 10/10; Videos S3E and S3F). In contrast, aged males that had just become sterile contained either no motile sperm (n = 7/10) or sperm showing limited motility (n = 3/10; Video S3G). Moreover, almost none of the females that had mated with these aged males contained motile sperm (1/10; Videos S3H and S3I). Therefore, *mzt1* mutant males become sterile due to the loss of sperm motility. Electron microscopy revealed no obvious structural defects in the axonemes of *mzt1* mutant sperm tails (Figures S4A and S4B), and the average number of cytoplasmic microtubules running through the sperm tails was similar between spermatids in *mzt1* mutant (29.9; n = 19) and wild-type (27.5; n = 13; t test; p = 0.83) testes. One of the mitochondrial derivatives, however, was sometimes severely misshapen in *mzt1* mutant spermatids (Figure S4A). Moreover, in testes from 4-week-old *mzt1* mutants, the plasma membranes of individualized mature spermatids were often separated from the mitochondrial derivatives and the two mitochondrial derivatives were frequently dissociated, unlike in wild-type testes (Figure S4C). The reason for these defects remains unclear, but disorganization of the sperm tails likely contributes to sperm motility defects in *mzt1* mutants.

We then questioned why Mzt1 may be required specifically in testes. The homologs of Mzt1 in *C. albicans* and cultured human cells help mediate interactions between the γ-TuRC and tethering proteins that contain a conserved CM1 domain close to their N terminus [9, 15]. The only reported CM1-containing gene in *Drosophila*, *cnn*, has multiple isoforms [31], including testes-specific isoforms that vary in both their N- and C-terminal regions (Figures 4A and 4B) [32, 33]. The main Cnn isoform, Cnn-PA, is expressed in most cell types and localizes to centrosomes, where it is essential for proper centrosome assembly and γ-TuRC recruitment [32, 34–36]. The testes-specific isoforms of Cnn (collectively called CnnT) have a modified C terminus that directs their localization to the mitochondrial derivatives (nebenkerns) in elongating spermatids [33]. In addition, the CnnT isoforms have a modified and shorter N-terminal region proximal to the CM1 domain (Figures 4A and 4B), and we hypothesized that Mzt1 may be necessary for the proper binding of these isoforms to γ-TuRCs. We found, however, that purified MBP-tagged N-terminal fragments of CnnT (MBP-CnnT-N) could bind equally well to γ-TuRCs in the presence or absence of Mzt1 (Figure 4C, lanes 3 and 4 in each panel). Moreover, Mzt1 was not present at nebenkerns in early-elongating spermatids (where CnnT recruits γ-TuRCs [33]) (Figures 4D–4F) and the localization of γ-tubulin, although strongly reduced at basal bodies, was unaffected at these nebenkerns in *mzt1* mutants (Figure 4G). We also found that Cnn was absent from centriole adjuncts (Figure 4H), where we have shown that Mzt1 is required for proper γ-tubulin localization (Figures 3I and 3J). Collectively, this shows that, in contrast to the homologs of Mzt1 and Cnn in *C. albicans* and cultured human cells [9, 15], *Drosophila* Mzt1 is not required for Cnn to bind γ-TuRCs. Our data do show, however, that Mzt1-dependent recruitment of γ-TuRCs is MTOC specific: Mzt1 is present in, and required for, γ-TuRCs that are recruited to basal bodies, but Mzt1 is absent from, and not required for, γ-TuRCs that are recruited to the nebenkerns in early-elongating spermatids. Thus, Mzt1 defines γ-TuRC heterogeneity within the same cell at the same developmental stage.

Intriguingly, although MBP-CnnT-N could bind γ-TuRCs, MBP-tagged N-terminal fragments of the main Cnn-PA isoform (MBP-Cnn-PA-N) failed to bind γ-TuRCs either with or without Mzt1 (Figure 4C, lanes 1 and 2 in each panel). Although this may be due to problems with protein folding, we suspect that it is actually due to the absence of post-translational modifications, such as phosphorylation, that are known to occur on Cnn-PA specifically at centrosomes [37]. Such regulation would not be unprecedented, as phosphorylation of the N-terminal region of yeast Spc110 (which contains a CM1 domain) regulates its ability to bind γ-TuRCs [38]. We speculate that the extra 58 amino acids at the extreme N-terminal end of Cnn-PA (compared to CnnT isoforms) may fold back and cover the CM1 domain until phosphorylation events relieve this inhibition, although this requires further investigation. Nevertheless, we propose that the altered N-terminal region of CnnT allows it to

Figure 2. Mzt1 Localization during Spermatogenesis Is Consistent with It Being a Member of the γ-TuRC

(A) Phase-contrast and fluorescent images of a whole testis expressing endogenously tagged sfGFP-Mzt1, which is almost entirely absent from the apical tip of the testis.

(B and C) Fluorescence and phase-contrast images of meiotic spermatocytes (yellow border, B), onion stage spermatids (white border, B and C), and an early-elongating round spermatid (orange border, C) in testes expressing sfGFP-Mzt1. Note that the fluorescence intensity of sfGFP-Mzt1 both in the cytoplasm and at the centrioles and/or basal bodies (arrow, spermatocyte; arrowheads, round spermatids; asterisk, early-elongating round spermatid) increases with progression from meiotic spermatocytes to early-elongating round spermatids.

(D) Fluorescence microscope images of centrioles and basal bodies from testes expressing endogenously tagged sfGFP-Mzt1 (green) and the centriole and basal body marker pUbq-RFP-PACT (red). Different stages of spermatogenesis are shown, and the images have been contrasted differently in order to visualize the relatively weak sfGFP-Mzt1 signal in spermatocytes (interphase, meiosis I, and meiosis II). The changing pattern of sfGFP-Mzt1 localization through development matches that expected for a γ-TuRC component.

(E) Confocal images of testes expressing endogenously tagged sfGFP-Mzt1 (green), immunostained for γ-tubulin (red) and Asl (magenta), and stained for DNA (blue). An intermediate (left) and a late-stage spermatid cyst (right) is shown. Images boxed in yellow are enlarged views of the centriole adjunct indicated by the arrowhead. Note that sfGFP-Mzt1 redistributes to the sperm tails in the late cyst (asterisks) when the centriole adjuncts are no longer present.

(F) Images show sperm bundles from live squashes of testes (in culture medium containing 100 μM colchicine) expressing γ-Tub23C-sfGFP (green) and TagRFP-T-Mzt1 (magenta). Boxes contain enlarged images to show the colocalization of γ-Tub23C-sfGFP and TagRFP-T-Mzt1 puncta.

(G and H) Fluorescence and phase-contrast images show the localization of sfGFP-Mzt1 and the centriole marker pUbq-RFP-PACT in meiotic spermatocytes either just before (G) or after (H) nuclear envelope breakdown. The majority of microtubules remain outside the nucleus, and some of these microtubules intercalate with the parafusorial membranes (phase dark), with which sfGFP-Mzt1 colocalizes. Note that sfGFP-Mzt1 does not seem to localize at chromosomes, as can appear in fixed samples (see Figures S2B–S2D).

See also Figures S1 and S2 and Video S2.

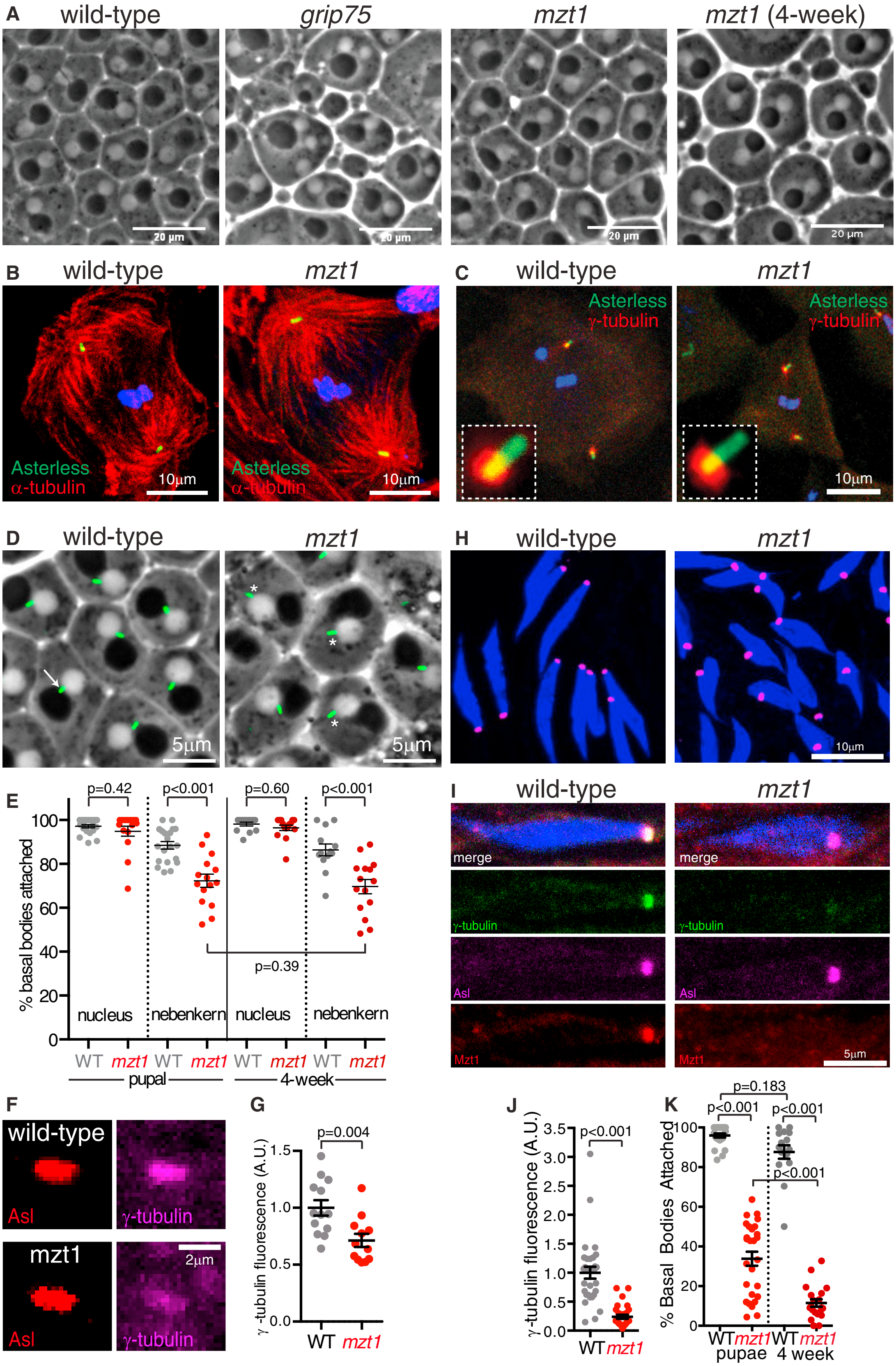

(legend on next page)

bind γ-TuRCs independently of centrosome-specific regulation, enabling CnnT to bind and recruit γ-TuRCs to mitochondria in spermatids.

The mechanism of Mzt1-dependent γ-TuRC recruitment to basal bodies remains unclear. It is possible that isoform differences in other γ-TuRC-tethering proteins, such as pericentrin-like protein (Plp) or ninein/Bsg25D (Nin), could confer a requirement for Mzt1 at basal bodies in the testes. Intriguingly, Nin isoforms vary in their N-terminal region, and γ-tubulin was recently shown to co-immunoprecipitate with an N-terminal fragment of Nin [39], but whether any Plp or Nin isoforms are testes specific remains unknown. Alternatively, Mzt1-dependent recruitment of γ-TuRCs may involve an as yet uncharacterized γ-TuRC-tethering protein that is basal body specific, or Mzt1 may function differently in flies from its homologs in yeast and human cells. It also remains unclear why the fertility of *mzt1* mutants decreases with age. We speculate that the age-related increase in basal body positioning defects could contribute, possibly because mispositioned basal bodies may lead to defects in axoneme movement, but this remains to be explored. It is also possible that the dedifferentiation of spermatogonia into stem cells during aging [40] is affected in *mzt1* mutants, although this is unlikely given that Mzt1 is not expressed in spermatogonia.

Most importantly, our work shows that different types of γ-TuRCs exist within the same organism and within the same cell and that γ-TuRC heterogeneity influences the recruitment of γ-TuRCs to specific MTOCs. The clear presence of γ-TuRC heterogeneity in flies is in agreement with recent observations in mouse keratinocytes, where the γ-TuRC-tethering protein NEDD1 binds only to γ-TuRCs that anchor, rather than nucleate, microtubules [41]. Thus, it is now becoming clear that heterogeneity in γ-TuRC composition is a genuine phenomenon that can influence γ-TuRC function. This may have medical implications, given that γ-TuRCs have been identified as potential anti-cancer targets [42–44], and in the future, it will be important to determine whether other γ-TuRC proteins confer γ-TuRC heterogeneity and how this may influence γ-TuRC function.

STAR★METHODS

Detailed methods are provided in the online version of this paper and include the following:

- KEY RESOURCES TABLE
- CONTACT FOR REAGENT AND RESOURCE SHARING
- EXPERIMENTAL MODEL AND SUBJECT DETAILS
  - *Drosophila melanogaster* stocks
- METHOD DETAILS
  - DNA cloning
  - Transgenic *Drosophila* lines
  - Primary Antibodies
  - Fertility tests
  - Yeast-two hybrid
  - Tissue expression analysis and western blotting
  - Recombinant protein expression and purification
  - Immunoprecipitation and Sucrose Gradient Sedimentation
  - Immunostaining
  - Light Microscopy
  - Transmission Electron Microscopy
- QUANTIFICATION AND STATISTICAL ANALYSIS

SUPPLEMENTAL INFORMATION

Supplemental Information includes four figures, one table, and three videos and can be found with this article online at https://doi.org/10.1016/j.cub.2018.05.044.

ACKNOWLEDGMENTS

This work was supported by a Wellcome Trust and Royal Society Sir Henry Dale Fellowship awarded to P.T.C. (105653/Z/14/Z) and a BBSRC DTP studentship awarded to C.A.T. (BB/M011194/1). We thank Simon Bullock,

Figure 3. Mzt1 Is Required for γ-TuRC Recruitment and Basal Body Positioning in Developing Sperm Cells, but Not for Male Meiosis

(A) Phase-contrast images show onion-stage round spermatid cysts from pupal wild-type, pupal *grip75* mutant, pupal *mzt1* mutant, or 4-week-old *mzt1* mutant flies, as indicated. Similar to wild-type cysts, but unlike *grip75* mutant cysts, *mzt1* mutant cysts normally have a 1:1 nucleus:nebenkern ratio and display little variation in nuclear size (see Figures S3A and S3B for quantification), indicating that meiosis proceeds normally.

(B and C) Confocal images show meiotic spermatocytes from either wild-type (left) or *mzt1* mutant (right) testes stained for DNA (blue) and immunostained for the centriole marker Asl (green) and either α-tubulin (B) or γ-tubulin (C) (red). Centrosomes in *mzt1* mutant meiotic spermatocytes can still organize robust microtubule asters (B) and can recruit large amounts of γ-tubulin (C; see Figure S3C for quantification).

(D and E) Fluorescence and phase-contrast images (D) and graph (E) show the results of an analysis of basal body positioning in onion-stage round spermatid cysts from testes of wild-type or *mzt1* mutant pupal or 4-week-old flies, as indicated, expressing the basal body marker pUbq-GFP-PACT. In (D), the arrow indicates a basal body that is correctly positioned between the nucleus and nebenkern in a wild-type cell, and the asterisks indicate basal bodies that are mispositioned in *mzt1* mutant cells.

(F and G) Confocal images (F) and graph (G) show that γ-tubulin recruitment to the basal bodies in round spermatids is strongly reduced in *mzt1* mutant round spermatids. The images in (F) show individual basal bodies from either wild-type (top) or *mzt1* mutant (bottom) testes immunostained for Asl (red) and γ-tubulin (magenta).

(H) Confocal images of intermediate spermatids from either wild-type (left) or *mzt1* mutant (right) testes immunostained with the centriole adjunct marker Asl (magenta) and stained for DNA (blue). Note that the centriole adjuncts in the *mzt1* mutant spermatids are not correctly positioned at the tips of nuclei.

(I and J) Confocal images (I) and graph (J) show that γ-tubulin recruitment to the centriole adjuncts is strongly reduced in *mzt1* mutant spermatids. The images in (I) show individual intermediate spermatids from either wild-type (left) or *mzt1* mutant (right) testes immunostained for Asl (magenta), γ-tubulin (green), and Mzt1 (red) and stained for DNA (blue). Mzt1 is present at the centriole adjunct in wild-type, but not *mzt1* mutant, spermatids.

(K) Graph shows the percentage of basal bodies per cyst attached to the basal tips of nuclei in pupal and 4-week-old wild-type or *mzt1* mutant testes, as indicated.

Each data point in (E), (G), (J), and (K) represents an average value from a cyst of cells. Error bars represent SEM. See also Figures S1, S3, and S4, Table S1, and Video S3.

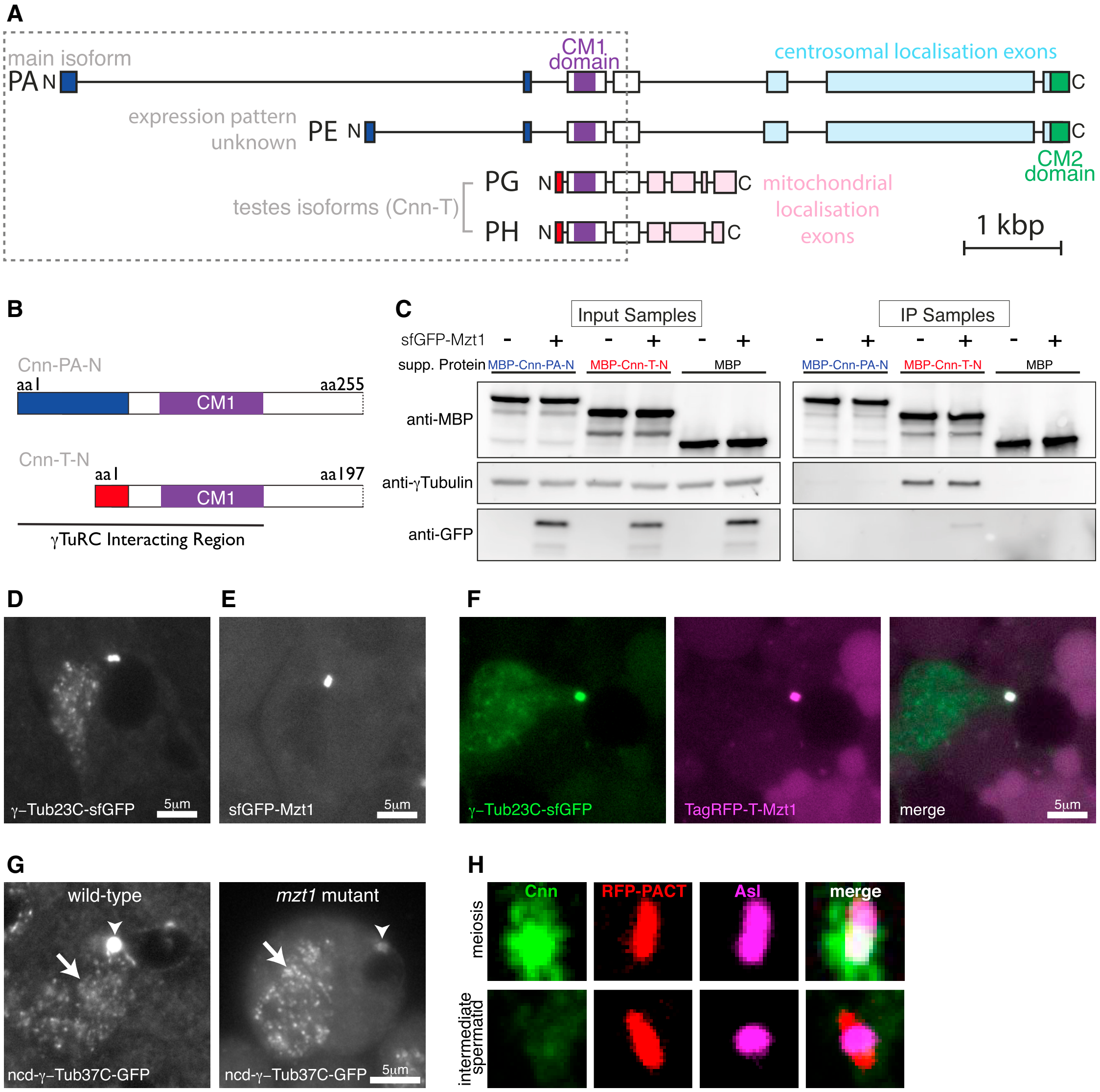

Figure 4. Centrosomin Functions Independently of Mzt1 in *Drosophila*

(A) The cartoon depicts the exon and intron map of several *cnn* isoforms; exons are shown as boxed regions and introns as lines. Only the *cnn* isoforms with full-length cDNA clones in flybase are shown. The C-terminal light blue exons in Cnn-PA (the main Cnn isoform) and Cnn-PE confer centrosomal localization, whereas the C-terminal pink exons in Cnn-PG and Cnn-PH (collectively known as Cnn-T) confer mitochondrial localization. The N-terminal dark-blue exons are specific to Cnn-PA and Cnn-PE, and the red exons are specific to Cnn-T isoforms.

(B) The cartoon depicts the N-terminal regions found in Cnn-PA (amino acids [aa] 1–255; Cnn-PA-N) and Cnn-T proteins (amino acids 1–197; Cnn-T-N) from the boxed region in (A) and that were used in the immunoprecipitation (IP) experiments in (C). The dark blue region is specific to Cnn-PA, and the red region is specific to Cnn-T.

(C) Western blots show the results of an anti-MBP co-immunoprecipitation experiment using MBP-tagged N-terminal fragments of Cnn-PA (amino acids 1–255; MBP-Cnn-PA-N) and Cnn-T (amino acids 1–197; MBP-Cnn-T-N) that were mixed with embryo extracts that either expressed (+) or did not express (–) sfGFP-Mzt1, as indicated. Note that γ-tubulin co-immunoprecipitated only with MBP-Cnn-T-N and equally well in the presence or absence of sfGFP-Mzt1.

(D–G) Images show early-elongating spermatids from squashes of live testes (incubated in 100 μM colchicine) expressing γ-Tub23C-sfGFP (D), sfGFP-Mzt1 (E), γ-Tub23C-sfGFP and TagRFP-T-Mzt1 (F), or ncd-γ-Tub37C-GFP (G) in either a wild-type (D–F; left panel in G) or a *mzt1* mutant (right panel in G) background. Arrowheads in (G) indicate centriole adjuncts at basal bodies, and arrows indicate the elongating mitochondrial derivatives. Note that γ-Tub23C-sfGFP, but not sfGFP-Mzt1 or TagRFP-T-Mzt1, is recruited to the elongating mitochondrial derivatives under these conditions and that the recruitment of ncd-γ-Tub37C-GFP to basal bodies, but not to the mitochondrial derivatives, is perturbed in *mzt1* mutants.

(H) Confocal images of testes expressing the centriole and basal body marker RFP-PACT immunostained for Cnn (green) and Asl (magenta), showing that Cnn is present at centrioles during meiosis but absent from the centriole adjunct at basal bodies in spermatids.

Filip Port, and Nadine Muschalik for CRISPR reagents and advice; Jordan Raff for the pUbq-GFP gateway vector, anti-MBP, and anti-Asl antibodies and purified MBP protein; Andrew Gillis for use of microscopes; Matt Wayland for help with confocal microscopy; Torsten Krude for advice and reagents for sucrose co-sedimentation experiments; Marc de la Roche for help with purifying MBP-tagged fragments; and Caroline Fabre, Alan

Wainman, and the members of the Conduit lab for critical reading of the manuscript.

AUTHOR CONTRIBUTIONS

P.T.C. and C.A.T. designed the study, performed experiments, analyzed data, and wrote the manuscript. P.T.C. made the CRISPR fly strains. C.E.T., E.H., Z.Z., A.E.D., and A.N.B. performed experiments under the supervision of P.T.C. and C.A.T. A.F. and E.J. prepared samples for EM and collected the EM images.

DECLARATION OF INTERESTS

The authors declare no competing interests.

Received: December 28, 2017
Revised: April 6, 2018
Accepted: May 16, 2018
Published: July 5, 2018

REFERENCES

1. Akhmanova, A., and Steinmetz, M.O. (2015). Control of microtubule organization and dynamics: two ends in the limelight. Nat. Rev. Mol. Cell Biol. *16*, 711–726.
2. Petry, S., and Vale, R.D. (2015). Microtubule nucleation at the centrosome and beyond. Nat. Cell Biol. *17*, 1089–1093.
3. Lin, T.C., Neuner, A., and Schiebel, E. (2015). Targeting of γ-tubulin complexes to microtubule organizing centers: conservation and divergence. Trends Cell Biol. *25*, 296–307.
4. Kollman, J.M., Merdes, A., Mourey, L., and Agard, D.A. (2011). Microtubule nucleation by γ-tubulin complexes. Nat. Rev. Mol. Cell Biol. *12*, 709–721.
5. Farache, D., Emorine, L., Haren, L., and Merdes, A. (2018). Assembly and regulation of γ-tubulin complexes. Open Biol. *8*, 170266.
6. Teixidó-Travesa, N., Roig, J., and Lüders, J. (2012). The where, when and how of microtubule nucleation - one ring to rule them all. J. Cell Sci. *125*, 4445–4456.
7. Hutchins, J.R.A., Toyoda, Y., Hegemann, B., Poser, I., Hériché, J.-K., Sykora, M.M., Augsburg, M., Hudecz, O., Buschhorn, B.A., Bulkescher, J., et al. (2010). Systematic analysis of human protein complexes identifies chromosome segregation proteins. Science *328*, 593–599.
8. Janski, N., Herzog, E., and Schmit, A.-C. (2008). Identification of a novel small Arabidopsis protein interacting with gamma-tubulin complex protein 3. Cell Biol. Int. *32*, 546–548.
9. Lin, T.-C., Neuner, A., Flemming, D., Liu, P., Chinen, T., Jäkle, U., Arkowitz, R., and Schiebel, E. (2016). MOZART1 and γ-tubulin complex receptors are both required to turn γ-TuSC into an active microtubule nucleation template. J. Cell Biol. *215*, 823–840.
10. Janski, N., Masoud, K., Batzenschlager, M., Herzog, E., Evrard, J.-L., Houlné, G., Bourge, M., Chabouté, M.-E., and Schmit, A.-C. (2012). The GCP3-interacting proteins GIP1 and GIP2 are required for γ-tubulin complex protein localization, spindle integrity, and chromosomal stability. Plant Cell *24*, 1171–1187.
11. Nakamura, M., Yagi, N., Kato, T., Fujita, S., Kawashima, N., Ehrhardt, D.W., and Hashimoto, T. (2012). Arabidopsis GCP3-interacting protein 1/MOZART 1 is an integral component of the γ-tubulin-containing microtubule nucleating complex. Plant J. *71*, 216–225.
12. Batzenschlager, M., Masoud, K., Janski, N., Houlné, G., Herzog, E., Evrard, J.-L., Baumberger, N., Erhardt, M., Nominé, Y., Kieffer, B., et al. (2013). The GIP gamma-tubulin complex-associated proteins are involved in nuclear architecture in Arabidopsis thaliana. Front. Plant Sci. *4*, 480.
13. Masuda, H., Mori, R., Yukawa, M., and Toda, T. (2013). Fission yeast MOZART1/Mzt1 is an essential γ-tubulin complex component required for complex recruitment to the microtubule organizing center, but not its assembly. Mol. Biol. Cell *24*, 2894–2906.
14. Dhani, D.K., Goult, B.T., George, G.M., Rogerson, D.T., Bitton, D.A., Miller, C.J., Schwabe, J.W.R., and Tanaka, K. (2013). Mzt1/Tam4, a fission yeast MOZART1 homologue, is an essential component of the γ-tubulin complex and directly interacts with GCP3(Alp6). Mol. Biol. Cell *24*, 3337–3349.
15. Cota, R.R., Teixidó-Travesa, N., Ezquerra, A., Eibes, S., Lacasa, C., Roig, J., and Lüders, J. (2017). MZT1 regulates microtubule nucleation by linking γTuRC assembly to adapter-mediated targeting and activation. J. Cell Sci. *130*, 406–419.
16. Chintapalli, V.R., Wang, J., and Dow, J.A.T. (2007). Using FlyAtlas to identify better Drosophila melanogaster models of human disease. Nat. Genet. *39*, 715–720.
17. Graveley, B.R., Brooks, A.N., Carlson, J.W., Duff, M.O., Landolin, J.M., Yang, L., Artieri, C.G., van Baren, M.J., Boley, N., Booth, B.W., et al. (2011). The developmental transcriptome of Drosophila melanogaster. Nature *471*, 473–479.
18. Tates, A.D. (1971). Cytodifferentiation during spermatogenesis in Drosophila melanogaster: an electron microscope study. PhD thesis (Rijksuniversiteit de Leiden).
19. White-Cooper, H. (2012). Tissue, cell type and stage-specific ectopic gene expression and RNAi induction in the Drosophila testis. Spermatogenesis *2*, 11–22.
20. Blachon, S., Gopalakrishnan, J., Omori, Y., Polyanovsky, A., Church, A., Nicastro, D., Malicki, J., and Avidor-Reiss, T. (2008). Drosophila asterless and vertebrate Cep152 Are orthologs essential for centriole duplication. Genetics *180*, 2081–2094.
21. Blachon, S., Cai, X., Roberts, K.A., Yang, K., Polyanovsky, A., Church, A., and Avidor-Reiss, T. (2009). A proximal centriole-like structure is present in Drosophila spermatids and can serve as a model to study centriole duplication. Genetics *182*, 133–144.
22. Fu, J., and Glover, D.M. (2012). Structured illumination of the interface between centriole and peri-centriolar material. Open Biol. *2*, 120104.
23. Vogt, N., Koch, I., Schwarz, H., Schnorrer, F., and Nüsslein-Volhard, C. (2006). The gammaTuRC components Grip75 and Grip128 have an essential microtubule-anchoring function in the Drosophila germline. Development *133*, 3963–3972.
24. Colombié, N., Vérollet, C., Sampaio, P., Moisand, A., Sunkel, C., Bourbon, H.-M., Wright, M., and Raynaud-Messina, B. (2006). The Drosophila γ-tubulin small complex subunit Dgrip84 is required for structural and functional integrity of the spindle apparatus. Mol. Biol. Cell *17*, 272–282.
25. Sampaio, P., Rebollo, E., Varmark, H., Sunkel, C.E., and González, C. (2001). Organized microtubule arrays in gamma-tubulin-depleted Drosophila spermatocytes. Curr. Biol. *11*, 1788–1793.
26. Barbosa, V., Gatt, M., Rebollo, E., Gonzalez, C., and Glover, D.M. (2003). Drosophila dd4 mutants reveal that gammaTuRC is required to maintain juxtaposed half spindles in spermatocytes. J. Cell Sci. *116*, 929–941.
27. Li, M.-G., Serr, M., Newman, E.A., and Hays, T.S. (2004). The Drosophila tctex-1 light chain is dispensable for essential cytoplasmic dynein functions but is required during spermatid differentiation. Mol. Biol. Cell *15*, 3005–3014.
28. Wei, H.-C., Rollins, J., Fabian, L., Hayes, M., Polevoy, G., Bazinet, C., and Brill, J.A. (2008). Depletion of plasma membrane PtdIns(4,5)P2 reveals essential roles for phosphoinositides in flagellar biogenesis. J. Cell Sci. *121*, 1076–1084.
29. Anderson, M.A., Jodoin, J.N., Lee, E., Hales, K.G., Hays, T.S., and Lee, L.A. (2009). Asunder is a critical regulator of dynein-dynactin localization during Drosophila spermatogenesis. Mol. Biol. Cell *20*, 2709–2721.
30. Sitaram, P., Anderson, M.A., Jodoin, J.N., Lee, E., and Lee, L.A. (2012). Regulation of dynein localization and centrosome positioning by Lis-1 and asunder during Drosophila spermatogenesis. Development *139*, 2945–2954.
31. Eisman, R.C., Phelps, M.A.S., and Kaufman, T.C. (2009). Centrosomin: a complex mix of long and short isoforms is required for centrosome

CellPress

function during early development in Drosophila melanogaster. Genetics *182*, 979–997.

32. Li, K., Xu, E.Y., Cecil, J.K., Turner, F.R., Megraw, T.L., and Kaufman, T.C. (1998). Drosophila centrosomin protein is required for male meiosis and assembly of the flagellar axoneme. J. Cell Biol. *141*, 455–467.

33. Chen, J.V., Buchwalter, R.A., Kao, L.-R., and Megraw, T.L. (2017). A splice variant of centrosomin converts mitochondria to microtubule-organizing centers. Curr. Biol. *27*, 1928–1940.e6.

34. Lucas, E.P., and Raff, J.W. (2007). Maintaining the proper connection between the centrioles and the pericentriolar matrix requires Drosophila centrosomin. J. Cell Biol. *178*, 725–732.

35. Zhang, J., and Megraw, T.L. (2007). Proper recruitment of gamma-tubulin and D-TACC/Msps to embryonic Drosophila centrosomes requires Centrosomin Motif 1. Mol. Biol. Cell *18*, 4037–4049.

36. Conduit, P.T., Brunk, K., Dobbelaere, J., Dix, C.I., Lucas, E.P., and Raff, J.W. (2010). Centrioles regulate centrosome size by controlling the rate of Cnn incorporation into the PCM. Curr. Biol. *20*, 2178–2186.

37. Conduit, P.T., Feng, Z., Richens, J.H., Baumbach, J., Wainman, A., Bakshi, S.D., Dobbelaere, J., Johnson, S., Lea, S.M., and Raff, J.W. (2014). The centrosome-specific phosphorylation of Cnn by Polo/Plk1 drives Cnn scaffold assembly and centrosome maturation. Dev. Cell *28*, 659–669.

38. Lin, T.-C., Neuner, A., Schlosser, Y.T., Scharf, A.N., Weber, L., and Schiebel, E. (2014). Cell-cycle dependent phosphorylation of yeast pericentrin regulates γ-TuSC-mediated microtubule nucleation. eLife *3*, e02208.

39. Zheng, Y., Mennella, V., Marks, S., Wildonger, J., Elnagdi, E., Agard, D., and Megraw, T.L. (2016). The Seckel syndrome and centrosomal protein Ninein localizes asymmetrically to stem cell centrosomes but is not required for normal development, behavior, or DNA damage response in Drosophila. Mol. Biol. Cell *27*, 1740–1752.

40. Cheng, J., Türkel, N., Hemati, N., Fuller, M.T., Hunt, A.J., and Yamashita, Y.M. (2008). Centrosome misorientation reduces stem cell division during ageing. Nature *456*, 599–604.

41. Muroyama, A., Seldin, L., and Lechler, T. (2016). Divergent regulation of functionally distinct γ-tubulin complexes during differentiation. J. Cell Biol. *213*, 679–692.

42. Cala, O., Remy, M.-H., Guillet, V., Merdes, A., Mourey, L., Milon, A., and Czaplicki, G. (2013). Virtual and biophysical screening targeting the γ-tubulin complex–a new target for the inhibition of microtubule nucleation. PLoS ONE *8*, e63908.

43. Whitehurst, A.W., Bodemann, B.O., Cardenas, J., Ferguson, D., Girard, L., Peyton, M., Minna, J.D., Michnoff, C., Hao, W., Roth, M.G., et al. (2007). Synthetic lethal screen identification of chemosensitizer loci in cancer cells. Nature *446*, 815–819.

44. Tillement, V., Haren, L., Roullet, N., Etievant, C., and Merdes, A. (2009). The centrosome protein NEDD1 as a potential pharmacological target to induce cell cycle arrest. Mol. Cancer *8*, 10.

45. Martinez-Campos, M., Basto, R., Baker, J., Kernan, M., and Raff, J.W. (2004). The Drosophila pericentrin-like protein is essential for cilia/flagella function, but appears to be dispensable for mitosis. J. Cell Biol. *165*, 673–683.

46. Hallen, M.A., Ho, J., Yankel, C.D., and Endow, S.A. (2008). Fluorescence recovery kinetic analysis of gamma-tubulin binding to the mitotic spindle. Biophys. J. *95*, 3048–3058.

47. Port, F., Chen, H.-M., Lee, T., and Bullock, S.L. (2014). Optimized CRISPR/Cas tools for efficient germline and somatic genome engineering in Drosophila. Proc. Natl. Acad. Sci. USA *111*, E2967–E2976.

48. Connolly, K., and Cook, R. (1973). Rejection responses by female Drosophila melanogaster: their ontogeny, causality and effects upon the behaviour of the courting male. Behaviour *44*, 142–166.

STAR★METHODS

KEY RESOURCES TABLE

REAGENT or RESOURCE	SOURCE	IDENTIFIER
Antibodies		
anti-Grip71 (rabbit polyclonal)	This study	N/A
anti-Mzt1 (rabbit polyclonal)	This study	N/A
anti-GFP (mouse monoclonal)	Roche	Cat# 11814460001, RRID:AB_390913
anti-γ-tubulin (mouse monoclonal)	Sigma-Aldrich/Merck	Cat# T5326, RRID:AB_532292
anti-Asl (guinea pig polyclonal)	Gift from Jordan Raff	N/A
anti-MBP (rabbit polyclonal)	Gift from Jordan Raff	N/A
anti-a-tubulin (mouse monoclonal)	Sigma-Aldrich/Merck	Cat# T9026, RRID:AB_477593
HRP-conjugated anti-mouse	Immunoreagents	Cat#GtxMu-003-DHRPX
HRP-conjugated anti-rabbit	Immunoreagents	Cat#GtxRb-003-DHRPX
Mouse Alexa Fluor 488 secondary	Abcam	Cat# ab150117, RRID:AB_2688012
Mouse Alexa Fluor 568 secondary	ThermoFisher	Cat# A-11031, RRID:AB_144696
Mouse Alexa Fluor 633 secondary	ThermoFisher	Cat# A-21052, RRID:AB_2535719
Rabbit Alexa Fluor 488 secondary	Abcam	Cat#Ab150081
Rabbit Alexa Fluor 568 secondary	ThermoFisher	Cat# A-11036, RRID:AB_10563566
Guinea Pig Alexa 488 secondary	ThermoFisher	Cat# A-11073, RRID: AB_2534117
Guinea Pig Alexa 568 secondary	ThermoFisher	Cat# A-11075, RRID:AB_2534119
Guinea Pig Alexa 633 secondary	ThermoFisher	Cat# A-21105, RRID:AB_2535757
Chemicals, Peptides, and Recombinant Proteins		
MBP-Cnn-T-N	This study	N/A
MBP-Cnn-PA	This study	N/A
MBP	Gift from Jordan Raff	N/A
YPD Agar	Sigma-Aldrich/Merck	Cat#Y1500
YPD broth	Sigma-Aldrich/Merck	Cat#Y1375
Adenosine hemisulphate salt	Sigma-Aldrich/Merck	Cat#A9126
Yeast nitrogen base without amino acids	Sigma-Aldrich/Merck	Cat#Y0626
Yeast synthetic drop-out medium supp. without leucine	Sigma-Aldrich/Merck	Cat#Y1376
Yeast synthetic drop-out medium supp. without tryptophan	Sigma-Aldrich/Merck	Cat#Y1876
Yeast synthetic drop-out medium supp. without leucine, tryptophan	Sigma-Aldrich/Merck	Cat#Y0750
Yeast synthetic drop-out medium supp. without leucine, tryptophan, histidine, adenine	Sigma-Aldrich/Merck	Cat#Y2021
X-alpha-Gal	Clontech	Cat#630463
Aureobasidin A	Clontech	Cat#630466
Colchicine	Sigma-Aldrich/Merck	Cat#C9754
Protease inhibitor cocktail	Sigma-Aldrich/Merck	Cat#P8340
Hoechst stain	Life Technologies	Cat#33342
Critical Commercial Assays		
Matchmaker Gold Yeast Two-Hybrid System	Clontech	Cat#630489
Mini prep kit	QIAGEN	Cat#27106
Gel extraction kit	QIAGEN	Cat#28704
Phusion master mix with HF buffer	NEB	Cat#F531
5-alpha competent *E. coli* (High Efficiency)	NEB	Cat#C2987I
HiFi mastermix	NEB	Cat#E2621
Experimental Models: Organisms/Strains		
S. cerevisiae: Y2HGold	Clontech	630498
S. cerevisiae: Y187	Clontech	630457

(*Continued on next page*)

Continued

REAGENT or RESOURCE	SOURCE	IDENTIFIER
D. melanogaster: w^{1118}	BDSC 3605	FBgn0003996
D. melanogaster: grip75^{175}	Gift from Jordan Raff	FBal0150800
D. melanogaster: grip75$^{DF(2L)ED8142}$	DGRC	150202
D. melanogaster: pUbq-GFP-PACT	[45]	N/A
D. melanogaster: pUbq-RFP-PACT	[36]	N/A
D. melanogaster: ncd-γ-Tub37C-GFP	[46]	N/A
D. melanogaster: pUbq-sfGFP-Mzt1	This study	N/A
D. melanogaster: pUbq-Mzt1	This study	N/A
D. melanogaster: sfGFP-Mzt1	This study	N/A
D. melanogaster: TagRFP-T-Mzt1	This study	N/A
D. melanogaster: gTub23C-sfGFP	This study	N/A
D. melanogaster: $mzt1^{11}$	This study	N/A
D. melanogaster: $mzt1^{57}$	This study	N/A
Oligonucleotides		
Screening for *mzt1* deletion: GGATGTCCAAGAACCAGCGTC	This study	N/A
Screening for mzt1 deletion: CAGGCCTGAGATTATGAAGGG	This study	N/A
Amplifying Cnn-PA fragment: GGGGACAAGTTTGTACAAAA AAGCAGGCTTAATGGACCAGTCTAAACAGGTTTTGCGGG	This study	N/A
Amplifying Cnn-PA fragment: GGGGACCACTTTGTACAAG AAAGCTGGGTTCTATAGGCGCTCGGCCAACATGAATTCC	This study	N/A
Amplifying Cnn-T fragment: GGGGACAAGTTTGTACAAAA AAGCAGGCTTAATGAATAGTAATCGAACGTCGTCTTCGC	This study	N/A
Amplifying Cnn-T fragment: GGGGACCACTTTGTACAAGA AAGCTGGGTTCTATAGGCGCTCGGCCAACATGAATTCC	This study	N/A
Screening for sfGFP: CTGAAGTTCATCTGCACCACC	This study	N/A
Screening for sfGFP: GCGGCGGTCACGAACTCCAGC	This study	N/A
Recombinant DNA		
pGBKT7 DNA-BD Vector	Clontech	630443
pGADT7 AD Vector	Clontech	630442
pGBKT7-53 Control Vector	Clontech	630489
pGBKT7-Lam	Clontech	630489
pDONR-pUbq-CnnPA	Gift from Jordan Raff	N/A
pDEST-hisMBP	Addgene	11085
pCFD3	Gift from S. Bullock	Addgene 49410
pCFD4	Gift from S. Bullock	Addgene 49411
pUbq (no tag) Gateway destination vector	Gift from Jordan Raff	N/A
pUGW (Gateway vector)	Gift from Jordan Raff	DGRC 1283
pBS-KS-attB1-2-PT-SA-SD-2-sfGFP-FlAsh-StrepII-TEV-3x-FLAG	DGRC	1314
pBS-KS-attB1-2-PT-SA-SD-2-TagRFP-T-3XHA	DGRC	1317
pBluescript SK+	Gift from S. Bullock	N/A
Software and Algorithms		
Fiji (ImageJ)	Open source	N/A
Prism	GraphPad	N/A
SnapGene	SnapGene	N/A
Other		
GFP_Trap beads	Chromotek	gtma; gta
Protein A Dynabeads	ThermoFisher	10001D
Voltelef oil 10S	VWR	24627.188

CONTACT FOR REAGENT AND RESOURCE SHARING

Further information and requests for resources and reagents should be directed to and will be fulfilled by the Lead Contact, Paul Conduit (ptc29@cam.ac.uk).

EXPERIMENTAL MODEL AND SUBJECT DETAILS

All fly strains were maintained at 18 or 25°C on Iberian fly food made from dry active yeast, agar, and organic pasta flour, supplemented with nipagin, propionic acid, pen/strep and food coloring.

Drosophila melanogaster stocks

The wild-type stock used was w^{1118}. For immunoprecipitation experiments, embryos were collected from either w^{1118} female flies or from female flies expressing a single copy of pUbq-sfGFP-Mzt1 or pUbq-Mzt1. All *mzt1* mutant experiments were carried out in either a $mzt1^{11}/mzt1^{57}$ or a $mzt1^{57}/mzt1^{57}$ background. All *grip75* mutant experiments were carried out in a $grip75^{175}/grip75^{DF(2L)ED8142}$ background. Experiments involving sfGFP-Mzt1, pUbq-GFP-PACT and/or pUbq-RFP-PACT were carried out in homozygous backgrounds. Experiments using TagRFP-T-Mzt1 and γ-Tub23C-sfGFP were carried out in heterozygous backgrounds (with a wild-type chromosome).

METHOD DETAILS

DNA cloning

5-alpha Competent *E. coli* (High Efficiency) (NEB) cells were used for bacterial transformations, DNA fragments were purified using QIAquick Gel Extraction Kits (QIAGEN), plasmid purification was performed using QIAprep Spin Miniprep Kits (Quiagen). Phusion High-Fidelity PCR Master Mix with HF Buffer (ThermoFisher Scientific) was used for PCRs.

Transgenic *Drosophila* lines

Fly lines expressing pUbq-RFP-PACT [36], pUbq-GFP-PACT [45] and ncd-γ-Tub37C-GFP [46] have been described previously. To make *mzt1* null mutant alleles, guide RNA sequences were cloned into the dual guide pCFD4 vector, as described in [47]. Two different vectors were generated, each containing guide RNAs complementary to the N- and C-terminal regions of the mzt1 coding sequence (line 1: U61: TCGTCGGATTTATGTTGTGT and U63: AAGTGATGATTCCGGAGAAT; line 2 U61: TGTTGATGCAGCCGA TTCTC and U63: CAGGATAGTGAAGCGATCGT); both C-terminal guides were designed to avoid cutting within the 3′UTR of CG3229, which overlaps the extreme C-terminal region of the *mzt1* coding sequence. These vectors were transformed into the attP40 landing site to generate guide-expressing flies that were then crossed to nos-Cas9-expressing females (Bloomington 54591). F1 males were outcrossed to balancer lines before being killed and screened by PCR (forward primer: CAGGCCTGAGATTA TGAAGGG; reverse primer: GGATGTCCAAGAACCAGCGTC). F2 male progeny from those F1 males carrying a germline deletion (based on a smaller PCR band) were outcrossed to balancer lines before being killed and screened by PCR. Balanced lines were produced from those F2 males that had incorporated the deletion allele. Two of the generated alleles were maintained: $mzt1^{11}$, which encodes the first four amino acids of Mzt1 followed by a scrambled sequence that terminates after 33 amino acids; and $mzt1^{57}$, which encodes the first ten amino acids followed directly by the last eight amino acids of Mzt1. To generate sfGFP- and Tag-RFP-T-tagged *mzt1* alleles, the guide RNA sequence (G)CAGGATAGTGAAGCGATCGT was cloned into the single guide pCFD3 vector, as described in [47], and the vector was transformed into the attP40 site. This line was then crossed to nos-Cas9 expressing females and the resulting embryos were injected with a pBluescript plasmid containing either the sfGFP or TagRFP-T tag and linker sequence (4X GlyGlySer) flanked on either side by 1.5kb of DNA homologous to the *mzt1* genomic locus surrounding the 5′ end of the coding region. This "homology" vector was made by HiFi assembly (NEB) of PCR fragments generated from genomic DNA prepared from nos-Cas-9 flies (using MicroLYSIS, Microzone) and a vector containing the sfGFP tag (DGRC, 1314) or the TagRFP-T tag (DGRC, 1317). F1 and F2 males were screened by PCR using primers specific to sfGFP (forward primer: CTGAAGTTCATCTGCACCACC; reverse primer: GCGGCGGTCACGAACTCCAGC). The endogenously tagged γ-Tub23C-sfGFP line was made in the same way, except that the guide RNA sequence (G)AGCGAACTAGGAACCGGCGC was used and the homology vector contained 1.5kb of DNA homologous to the *γ-tubulin23c* genomic locus surrounding the 3′ end of the coding region. To generate pUbq-sfGFP-Mzt1 and pUbq-Mzt1 lines, sfGFP-Mzt1 or Mzt1 were cloned into the pUbq Gateway transformation vector (gift from J. Raff) using HiFi assembly of PCR amplified products. All DNA vectors were injected into embryos by the Department of Genetics Fly Facility, Cambridge, UK.

Primary Antibodies

Affinity purified rabbit polyclonal antibodies raised against the Grip71 peptide sequence RKPQPYETANRQSLC or the Mzt1 peptide sequence SEQPTQHKDDRFT were generated by Cambridge Research Biochemicals. For western blotting, the following antibodies were used: anti-GFP mouse monoclonal at 1:250 or 1:500 (Roche, 11814460001), anti-γ-Tubulin mouse monoclonal at 1:500 (Sigma, GTU-88), anti-Grip71 rabbit polyclonal at 1:100 (this study), an N-terminal anti-Asl guinea pig polyclonal at 1:1000 (Gift from Jordan Raff) and anti-MBP rabbit polyclonal at 1:3000 (gift from Jordan Raff). For immunostaining, the following antibodies

were used: anti-γ-Tubulin mouse monoclonal at 1:500 (Sigma, GTU-88), anti-Asl guinea pig polyclonal at 1:1000 (Gift from Jordan Raff), anti-α-tubulin mouse monoclonal at 1:1000 (Sigma, DM1a), and anti-Mzt1 rabbit polyclonal at 1:100 (this study). DNA was stained with Hoechst (Life Technologies, 33342).

Fertility tests

Cages that were sealed with apple juice agar plates with a spot of dried yeast paste were set up at 25°C containing between ~30 and ~50 newly-hatched test flies (e.g., male *mzt1*^{11}/*mzt1*^{57}) and ~50 newly-hatched wild-type virgin females (except when testing *mzt1* mutant females). The wild-type flies were replaced each week with newly-hatched virgins to control for the effect of their aging and to ensure that hatching rates were dependent on the females mating with males of the correct age (females can store sperm and tend to reject males once they have already mated [48]). The apple juice agar plates were exchanged with fresh plates 2-4 times a day, and the removed plates were kept at 25°C for at least 25 hours before the proportion of hatched eggs was calculated. A minimum number of 30 eggs were counted, and if egg numbers on a single plate were below 30 then two or more consecutive counts were added together. The total number of counts each week for each cross used to generate the data in Figure 1F was as follows: Week 1: WT males x WT females, n = 14; *mzt1* males x WT females, n = 14; sfGFP-Mzt1 males x WT females, n = 14; WT males x *mzt1* females, n = 15; Week 2: WT males x WT females, n = 9; *mzt1* males x WT females, n = 10; sfGFP-Mzt1 males x WT females, n = 11; WT males x *mzt1* females, n = 15; Week 3: WT males x WT females, n = 10; *mzt1* males x WT females, n = 4; sfGFP-Mzt1 males x WT females, n = 12; WT males x *mzt1* females, n = 16; Week 4: WT males x WT females, n = 7; *mzt1* males x WT females, n = 2; sfGFP-Mzt1 males x WT females, n = 7; WT males x *mzt1* females, n = 7.

Yeast-two hybrid

Full-length Mzt1 was cloned into the pGBKT7 vector (Clontech) and N-terminal fragments of *grip84* (aa1-274), *grip91* (aa1-231), *grip128* (aa1-282) and *grip163* (aa1-256) were cloned into the pGADT7 vector (Clontech); the plasmids were transfected into either Y2HGold yeast (bait) or Y187 yeast (prey) (Clontech) and the transformed yeast was grown on single dropout (SDO) medium (containing a nitrogen base, a carbon source and a dropout supplement that contained specific amino acids and nucleosides). SDO medium lacked either tryptophan (for yeast containing pGBKT7-Mzt1) or leucine (yeast containing pGADT7 + NTE fragment). The bait and prey yeast strains were then mated following the guidelines in the Matchmaker Gold user manual (Clontech). The concentration of mated diploid cells was calculated on a spectrophotometer and appropriate dilutions were made before spot plating onto selection plates. Double dropout (DDO) plates lacked tryptophan and leucine in order to select for the bait and prey plasmids, while quadruple dropout (QDO) plates lacked tryptophan, leucine, histidine and adenine, and also contained the antibiotic aureobasidin A, in order to select for bait and prey interactions. Both plate types also contained X-α-Gal, which generates a blue color when interactions occur. Note that the plates contain a low concentration of adenine and that the yeast contain a mutation in the ADE2 gene; this means that in the absence of interaction the yeast attempt to make their own adenine and generate an intermediate red pigment, AIR, that perturbs yeast growth, such that yeast containing fragments that don't interact grow poorly even on DDO medium. Plates were incubated at 30°C for ~3 days before images were taken on a digital camera.

Tissue expression analysis and western blotting

Tissues from homozygous sfGFP-Mzt1 flies were dissected in PBS, transferred to 2x Laemmli Sample Buffer (BioRad) with β-mercaptoethanol (1:20), homogenized with forceps and the samples were denatured at 95°C for 10 minutes. Samples were run on a 4%–20% TGX Precast Gel (BioRad), alongside 5μl Precision Plus WesternC Standard markers (BioRad). Semi-dry western blotting was carried out using TransBlot Turbo 0.2μm nitrocellulose membrane transfer packs (BioRad), and a TransBlot Turbo transfer system running at 1.3A, up to 25V, for 7 minutes. Membranes were stained with Ponceau, washed, first with distilled water then with milk solution (PBS + 0.1% triton + 4% milk powder), and then blocked in milk solution for 1 hour at room temperature. Sections of the blots were incubated with primary antibodies (as indicated in Figure 1D) overnight at 4°C. Blots were incubated with horseradish peroxidase-conjugated secondary antibodies (ImmunoReagents, 1:2000) for 45 mins at room temperature) and then with ECL substrate (BioRad) for 5 minutes at room temperature. Membranes were imaged on a Kodak Image Station 4000R.

Recombinant protein expression and purification

To generate a Cnn-T-specific N-terminal region of Cnn, we synthesized and PCR amplified an appropriate DNA fragment (made by Genewiz, based on the sequence of Cnn-T in flybase, and used it to replace the N-terminal region of Cnn in a pDONR-Cnn-PA vector cut with XmaI. Fragments of Cnn-PA and Cnn-T encoding the N-terminal 255 and 197 amino acids, respectively, were amplified by PCR (Cnn-PA fragment forward primer: GGGGACAAGTTTGTACAAAAAAGCAGGCTTAATGGA

CCAGTCTAAACAGGTTTTGCGGG, reverse primer: GGGGACCACTTTGTACAAGA

AGCTGGGTTCTATAGGCGCTCGGCCAACATGAATTCC, Cnn-T fragment forward primer: GGGGACAAGTTTGTACAAAAAAGC AGGCTTAATGAATAGTAATCGAACG

TCGTCTTCGC, reverse primer: GGGGACCACTTTGTACAAGAAAGCTGGGTTCT

ATAGGCGCTCGGCCAACATGAATTCC) and inserted into a pDEST-HisMBP (Addgene, #11085) vector by Gateway cloning (Thermo Fisher Scientific). Proteins were expressed in *Escherichia coli* (BL21-DE3) and purified using affinity chromatography (gravity flow through amylose resin, New England Biolabs) and step elution in maltose. The concentration of each fraction was determined on a Nanodrop and peak fractions were diluted 1:1 with glycerol and stored at −20°C.

Immunoprecipitation and Sucrose Gradient Sedimentation

1g/ml of embryos were homogenized with a hand-pestle in homogenization buffer containing 50 mM HEPES, pH7.6, 1mM $MgCl_2$, 1 mM EGTA, 50 mM KCl and the protease inhibitors PMSF, Protease Inhibitor Cocktail (Sigma Aldrich) and DTT. Extracts were clarified by centrifugation twice for 15 minutes at 16,000 *rcf.* at 4°C.

For immunoprecipitation, the clarified embryo extract was diluted 2-fold with homogenization buffer, and an input sample was taken. 200 µL of this diluted extract was used per IP. 50 µL per IP of GFP-Trap beads (Chromotek) were equilibrated as per the manufacturer's instructions, and were rotated with the embryo extract overnight at 4°C. A sample of the unbound extract was taken and the beads were washed 5X 10 minutes at 4°C in PBT. The bound protein was eluted in 2x Laemmli Sample Buffer by boiling at 95°C for 10 minutes and a western blot was performed as above. For the MBP-Cnn fragment IPs, 200 µL of diluted extract was supplemented with purified MBP-Cnn fragments (to a final concentration of 2.5 µg/ml) and rotated at 4°C for 1 hour. Input samples were taken and the remaining extract was incubated with 30 µL magnetic protein A dynabeads (Life Technologies) coupled to anti-MBP antibodies (gift from Jordan Raff) at 4°C overnight. Beads were washed in PBT, boiled in sample buffer, and separated from the sample using a magnet. Input samples and IP samples were analyzed by western blotting as described above.

For sucrose gradient sedimentation, 90 µL of undiluted clarified extract was loaded onto a 4.75ml sucrose gradient (5%–40% w/v sucrose in homogenization buffer, made in 5x950 µL steps and allowed to diffuse into a continuous gradient overnight at 4°C) and centrifuged at 225,000 g for 4 hours 45 minutes at 4°C in a Beckman MLS 50 rotor. Fractions of 250 µL were taken from the top of the gradient, and the pellet was resuspended in 250 µL 40% sucrose. All fractions were analyzed by western blotting as above. Band intensities were quantified using Fiji (ImageJ) and each protein's intensity was normalized to its median.

Immunostaining

Testes were dissected in PBS, fixed in 4% paraformaldehyde for 30 minutes, washed 3X 5 minutes in PBS and incubated in 45% and then 60% acetic acid before being squashed onto slides and flash-frozen in liquid nitrogen (the acetic acid steps were omitted if fluorescent tags were expressed in the testes). Coverslips were removed and samples were post-fixed in methanol at −20°C, washed 3X 15 minutes in PBS + 0.1% Triton (PBT), then incubated overnight in a humid chamber at 4°C with primary antibodies diluted in PBT + 5% BSA + 0.02% azide. Slides were washed 3x 5 minutes in PBT and then incubated for 2 hours at room temperature with Alexa Fluor secondary antibodies (ThermoFisher) (all 1:1000 in PBT + 5% BSA + 0.02% azide). Slides were washed 3x 15 minutes in PBT, 10 minutes in PBT with Hoechst, and then 5 minutes in PBT. 10 µL of mounting medium (85% glycerol in H_20 + 2.5% N-propyl-galate) was placed on top of the tissue and a coverslip was gently lowered and sealed with nail varnish. Antennae were dissected in PBS, fixed in 4% paraformaldehyde for 20 minutes, washed 3X in PBS and then squashed between a slide and a coverslip in mounting medium.

Light Microscopy

Confocal fluorescence microscopy was performed on a Leica SP5 point scanning upright confocal system run by LAS AF software using a 63X 1.3NA glycerol objective (Leica 1156194). Epifluorescence microscopy and phase contrast microscopy were performed on a Leica DM IL LED inverted microscope controlled by µManager software and coupled to a RetigaR1 monochrome camera (QImaging) and a CoolLED pE-300 Ultra light source using either a 10X 0.22NA air objective (Leica 11506271), 20X 0.3NA air objective (Leica 11506272), a 40X 0.55NA air objective (Leica 11506298) or a 63X 1.3NA oil objective (Leica 11506384). All images were processed using Fiji (ImageJ).

For analysis of round spermatids under phase contrast, testes were dissected in PBS, transferred to a 50 µL droplet of PBS on a slide, cut open midway along the testes and, under observation, gently squashed under a coverslip using blotting paper; images were taken using a 40X 0.55NA air objective. For analysis of sperm motility, testes and the associated seminal vesicle were dissected in PBS and processed as above, except that the cut was performed at the junction between the testes and the seminal vesicle and the sperm were observed using 20X 0.3NA or 40X 0.55NA air objectives. For examining sfGFP-Mzt1, TagRFP-T-Mzt1 and g-Tub23C-sfGFP localization in sperm tails, testes were processed as above except that they were squashed in Schneider's insect medium supplemented with FBS, Pen/Strep and 100 µM colchicine. For analysis of nuclei distribution in sperm bundles, testes were processed as above except that they were squashed in Schneider's insect medium supplemented with Hoechst. For analysis of sfGFP-Mzt1 localization in a RFP-PACT background in Figure 2D, testes from pupae were dissected in Voltalef Oil 10S (VWR) on a glass bottom microwell MatTek dish (P35G-1.5-14-C), spread across the glass using forceps and observed using a 63X 1.3NA oil objective.

Transmission Electron Microscopy

Testes were dissected in PBS and fixed in a solution of 2.5% glutaraldehyde, 2% PFA, and 0.1M PIPES (pH7.2), at room temperature for one hour and then overnight at 4°C. They were washed 3x 20min in 0.1M PIPES buffer, 1x 20 min in 0.1M PIPES with 50mM glycine, and then 1x 20 min with 0.1M PIPES, all with rotation. The testes were secondary fixed by incubating 1x 2hrs at 4°C with vigorous rotation in 0.1 M PIPES containing 1% osmium tetroxide and then rinsed 5x 10min in MQ water. The testes were tertiary fixed in 0.5% uranyl acetate overnight at 4°C in the dark and then rinsed 3x 10min in MQ water. They were then step dehydrated in 30%, 50%, 70%, 80%, 90%, 95% ice-cold ethanol, each for 15 mins at 4°C with rotation, and then 3x 90 mins in 100% ice-cold ethanol at 4°C with rotation. For epoxy resin infiltration, the testes were incubated for 1-2 hr in 25% Agar 100 resin (Agar Scientific) in pure ethanol, for 2-3 hr in 50% Agar 100 in pure ethanol, for 1 hr in 75% Agar 100 in pure ethanol, and then overnight

in 100% Agar 100, all with rotation. The samples were carefully transferred into fresh 100% Agar 100 resin, and the resin was changed 3 times over the course of 2 days. The testes were placed in embedding molds on top of a thin layer of pre-polymerized Agar 100 and the molds were filled with fresh Agar 100 resin and incubated overnight at 60°C. Ultrathin sections (90 nm) were cut on a microtome and transfered to 50 mesh formvar coated copper grids (Agar Scientific) and the grids were post-stained with lead citrate for 5 min. The sections were imaged on a FEI Tecnai transmission electron microscope operated at 120kV with up to 30 degree tilt where required. Digital images were acquired using a Gatan Oneview camera.

QUANTIFICATION AND STATISTICAL ANALYSIS

Statistical analysis and graph production were performed using GraphPad Prism 6 or 7. Datasets were tested for normality by using D'Agostino-Pearson omnibus normality tests and subsequent statistical tests were chosen accordingly. A one-way ANOVA corrected for multiple comparisons (Dunnett test) was used to compare nucleus:nebenkern ratios and variations in nuclear diameter between wild-type and mutant round spermatids (Figures S3A and S3B). N numbers were as follows: wild-type, 30 cysts from 9 flies; *grip75* mutant, 29 cysts from 9 flies; *mzt1* mutant, 24 cysts from 14 flies; 4-week *mzt1* mutant, 29 cysts from 11 flies. Mann-Whitney tests were used to compare the γ-tubulin fluorescence at centrosomes in spermatocytes (Figure S3C). Each data point on the graph refers to an individual testis. To generate these data points, multiple images that each contained multiple centrosomes were taken from individual testes. An average centrosomal intensity for each image was obtained and the mean value of multiple images from a single testis was used to generate an average centrosomal intensity for each testis. N numbers were as follows: wild-type interphase, 14 testes examined with an average of 2.7 images per testis and 11.3 centrosomes per image; wild-type meiosis, 12 testes examined with an average of 2.1 images per testis and 7.6 centrosomes per image; *mzt1* mutant interphase, 12 testes examined with an average of 2.4 images per testis and 9.2 centrosomes per image; *mzt1* mutant meiosis, 10 testes examined with an average of 2.2 images per testis and 8.9 centrosomes per image. A one-way ANOVA with correction for multiple comparisons (Sidak test) was used to compare basal body attachment in round spermatids and intermediate spermatids (Figures 3E and 3K). N numbers for round spermatids were as follows: wild-type pupal, 662 basal bodies from 20 cysts from 7 testes; *mzt1* mutant pupal, 626 basal bodies from 15 cysts from 7 testes; wild-type 4-week, 429 basal bodies from 12 cysts from 12 testes; *mzt1* mutant 4-week, 590 basal bodies from 15 cysts from 9 testes. N numbers for intermediate spermatids were as follows: wild-type pupal, 884 basal bodies from 24 cysts from 9 testes; *mzt1* mutant pupal, 1015 basal bodies from 27 cysts from 10 testes; wild-type 4-week, 422 basal bodies from 15 cysts from 7 testes; *mzt1* mutant 4-week, 714 basal bodies from 20 cysts from 7 testes. A t test was used to compare γ-tubulin recruitment to basal bodies (in round spermatids, Figure 3G) and centriole adjuncts (in intermediate spermatids, Figure 3J) with the following N numbers for round spermatids: wild-type, 135 basal bodies from 13 cysts from 4 testes; *mzt1* mutant, 319 centriole adjuncts from 12 cysts from 6 testes. For intermediate spermatids: wild-type, 933 centriole adjuncts from 22 cysts from 9 testes; *mzt1* mutant, 1093 centriole adjuncts from 21 cysts from 9 testes. Protein alignments were produced using JalView.

Current Biology, Volume *28*

Supplemental Information

γ-TuRC Heterogeneity Revealed by Analysis of Mozart1

Corinne A. Tovey, Chloe E. Tubman, Eva Hamrud, Zihan Zhu, Anna E. Dyas, Andrew N. Butterfield, Alex Fyfe, Errin Johnson, and Paul T. Conduit

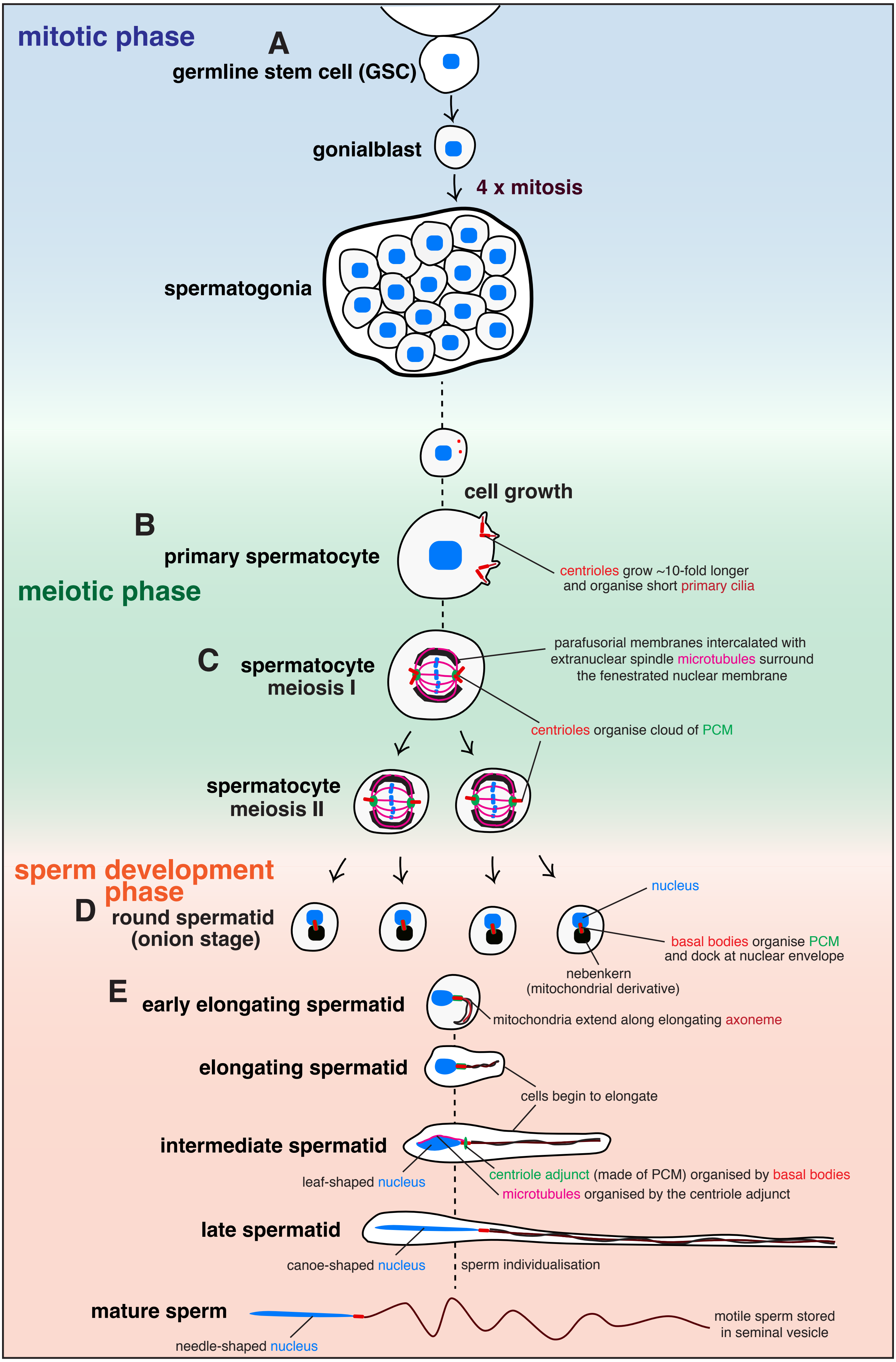
mitotic phase
A
germline stem cell (GSC)
gonialblast
4 x mitosis
spermatogonia
cell growth
B
primary spermatocyte
centrioles grow ~10-fold longer and organise short primary cilia
meiotic phase
C
spermatocyte meiosis I
parafusorial membranes intercalated with extranuclear spindle microtubules surround the fenestrated nuclear membrane
centrioles organise cloud of PCM
spermatocyte meiosis II
sperm development phase
D
round spermatid (onion stage)
nucleus
basal bodies organise PCM and dock at nuclear envelope
nebenkern (mitochondrial derivative)
E
early elongating spermatid
mitochondria extend along elongating axoneme
elongating spermatid
cells begin to elongate
intermediate spermatid
leaf-shaped nucleus
centriole adjunct (made of PCM) organised by basal bodies
microtubules organised by the centriole adjunct
late spermatid
canoe-shaped nucleus
sperm individualisation
mature sperm
needle-shaped nucleus
motile sperm stored in seminal vesicle

Figure S1. Stages of spermatogenesis in *Drosophila*, related to Figures 2 and 3. Cartoon summarises the various stages of spermatogenesis in *Drosophila*. (**A**) Spermatogenesis starts when a germline stem cell divides to produce a gonialblast. Gonialblasts go through four rounds of synchronous mitosis to produce cysts of 16 interconnected spermatogonia. (**B**) Each cell matures and grows in size to become a primary spermatocyte. The centrioles (red) increase ~10-fold in length and become resolvable by standard-resolution light microscopy. They are positioned at the cell cortex during interphase and nucleate short primary cilia. (**C**) As the cells enter meiosis the centrioles migrate to the nuclear envelope and recruit large amounts of PCM, which contains γ-TuRCs that nucleate large numbers of cytoplasmic microtubules. The nuclear envelope partially breaks down close to the centrosomes and spindle microtubules penetrate the nucleus; most spindle microtubules, however, remain outside the nuclear envelope and intercalate with a series of double membranes called the parafusorial membranes. (**D**) After the second meiotic division, the centrioles, soon to become basal bodies, are inherited by the round spermatid cells and become anchored at the nuclear envelope, positioned between the nucleus and mitochondrial derivative (nebenkern) in a Dynein-dependent manner. The spermatids remain as 64-cell cysts until sperm individualisation at the end of spermatogenesis. (**E**) In early elongating spermatids, the two mitochondrial derivatives from the nebenkern extend and wrap around the axoneme, which grows from the basal body. The cells begin to elongate and in intermediate spermatids the PCM is reorganised into the centriole adjunct. The basal body embeds into an indentation of the nuclear envelope called the nuclear socket at the basal tip of the nucleus, and cytoplasmic microtubules emanate from the centriole adjunct and run along the outer nuclear membrane to form part of the dense complex. Microtubules are also nucleated by γ-TuRCs that are recruited to the surface of the mitochondrial derivatives. In late spermatids the centriole adjunct dissipates, the nuclei progressively become needle-like in shape, and the cells are eventually separated into individual sperm; they become motile and are stored in the seminal vesicles. The mitochondrial derivatives persist along the length of the sperm tails throughout sperm differentiation.

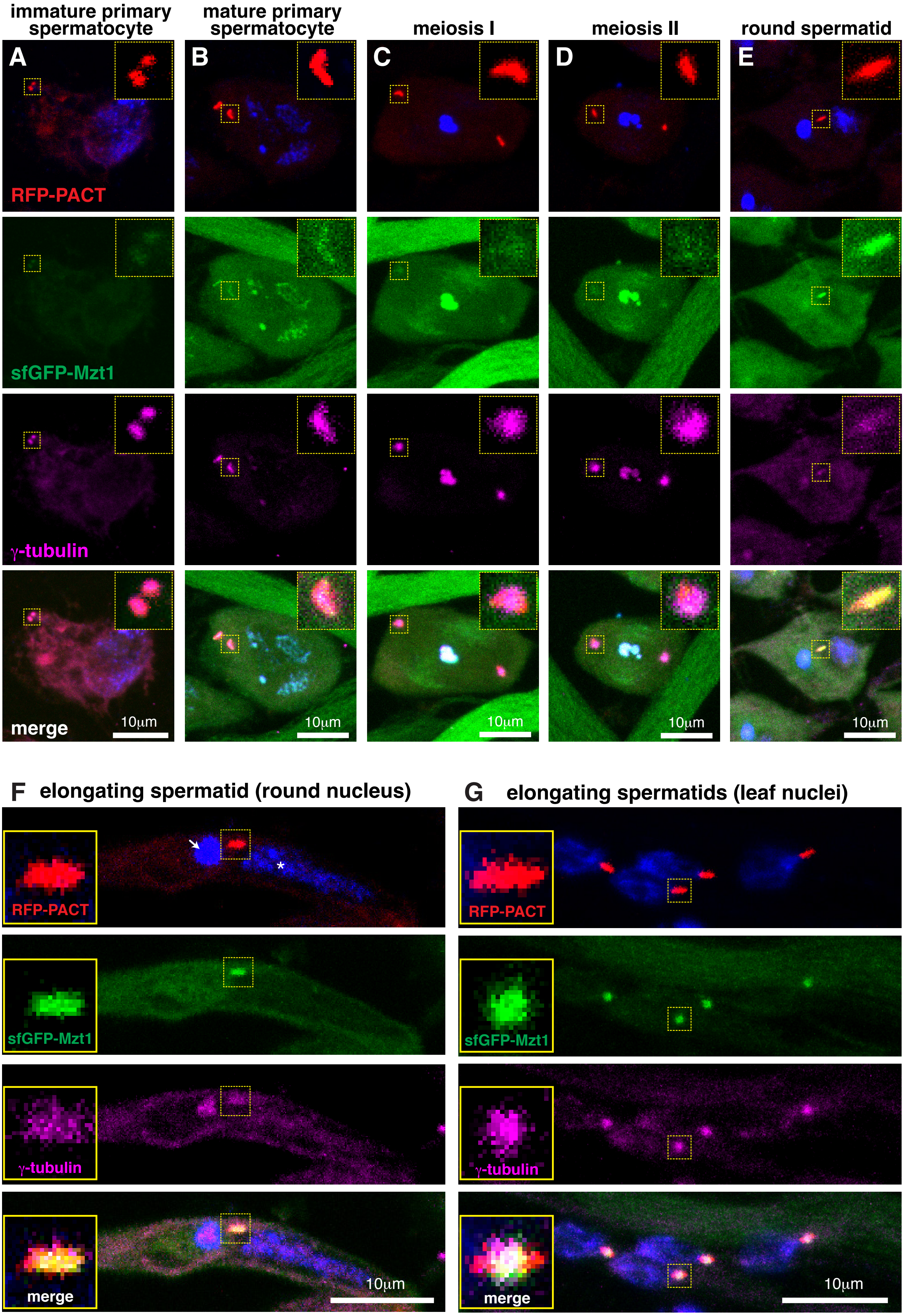

immature primary spermatocyte
mature primary spermatocyte
meiosis I
meiosis II
round spermatid
A
B
C
D
E
RFP-PACT
sfGFP-Mzt1
γ-tubulin
merge
10μm
F elongating spermatid (round nucleus)
G elongating spermatids (leaf nuclei)
RFP-PACT
sfGFP-Mzt1
γ-tubulin
merge
10μm

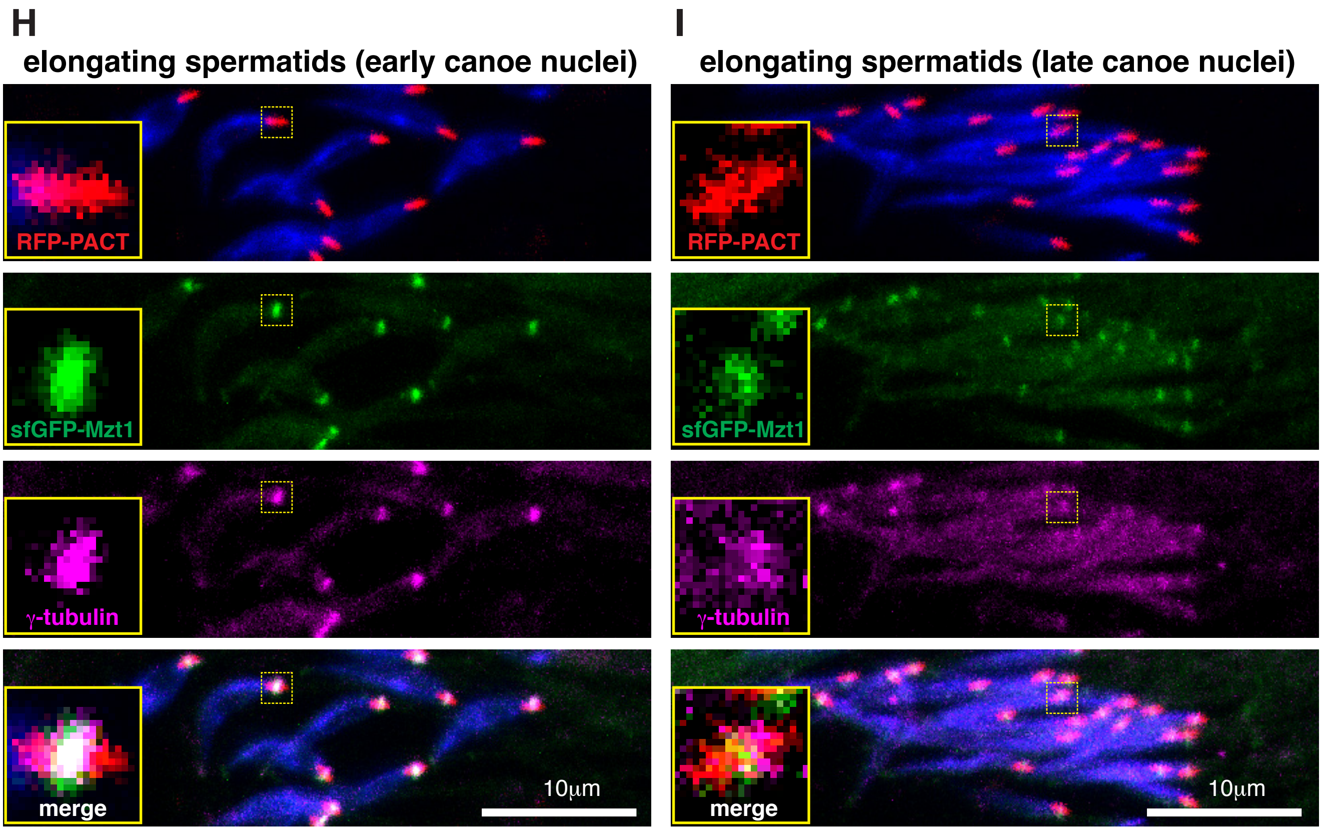
H
elongating spermatids (early canoe nuclei)
RFP-PACT
sfGFP-Mzt1
γ-tubulin
merge
10μm
I
elongating spermatids (late canoe nuclei)
RFP-PACT
sfGFP-Mzt1
γ-tubulin
merge
10μm

Figure S2. Analysis of γ-tubulin and sfGFP-Mzt1 localisation during spermatogenesis from spermatocytes to round spermatids, related to Figure 2. Testes expressing sfGFP-Mzt1 (green) and RFP-PACT (red) were fixed and stained for γ-tubulin (magenta) and DNA (blue). RFP-PACT marks centrioles and basal bodies. Images of cells at different developmental stages (as indicated at the top of each panel) are displayed in temporal order (earliest stage on the left); note that images of spermatogonial cells, where sfGFP-Mzt1 is not detected, are not shown due to space constraints. The centrosomes (A-D) or basal bodies (E-I) are enlarged in the yellow boxes in each image. (**A**) sfGFP-Mzt1 is present at very low levels in immature spermatocytes but does co-localise with γ-tubulin at centrioles. (**B**-**D**) sfGFP-Mzt1 is present at higher levels in mature spermatocytes and co-localises with γ-tubulin at the centrioles. Note that whereas the γ-tubulin is strongly concentrated around the centrioles in meiotic spermatocytes, the sfGFP-Mzt1 signal is relatively weak (**C**,**D**). Note also that the bright sfGFP-Mzt1 signal at the chromatin in (C) and (D) is likely due to "bleed through" from the Hoechst signal, as we do not observe it in live specimens (see Figure 2G,H). (**E**) sfGFP-Mzt1 concentrates strongly at basal bodies in round spermatids, co-localising with a relatively weak signal of γ-tubulin. (**F**) Images show a very early elongating spermatid where the nucleus (arrow) is still round but the mitochondrial derivatives (asterisk) have started to elongate along the sperm tail. sfGFP-Mzt1 and γ-tubulin localise along the length of the basal body. (**G**) Images show a group of early spermatids, as judged by the leaf-shape nuclei. sfGFP-Mzt1 and γ-tubulin localise at the centriole adjunct, which forms a ring around the centre of the basal body. (**H**) Images show a group of intermediate spermatids where the nuclei have become canoe-shaped. sfGFP-Mzt1 and γ-tubulin still colocalise at the centriole adjunct. (**I**) Images show a group of more mature spermatids where the nuclei have elongated further (late canoe nuclei) and both sfGFP-Mzt1 and γ-tubulin have started to dissipate from the basal body.

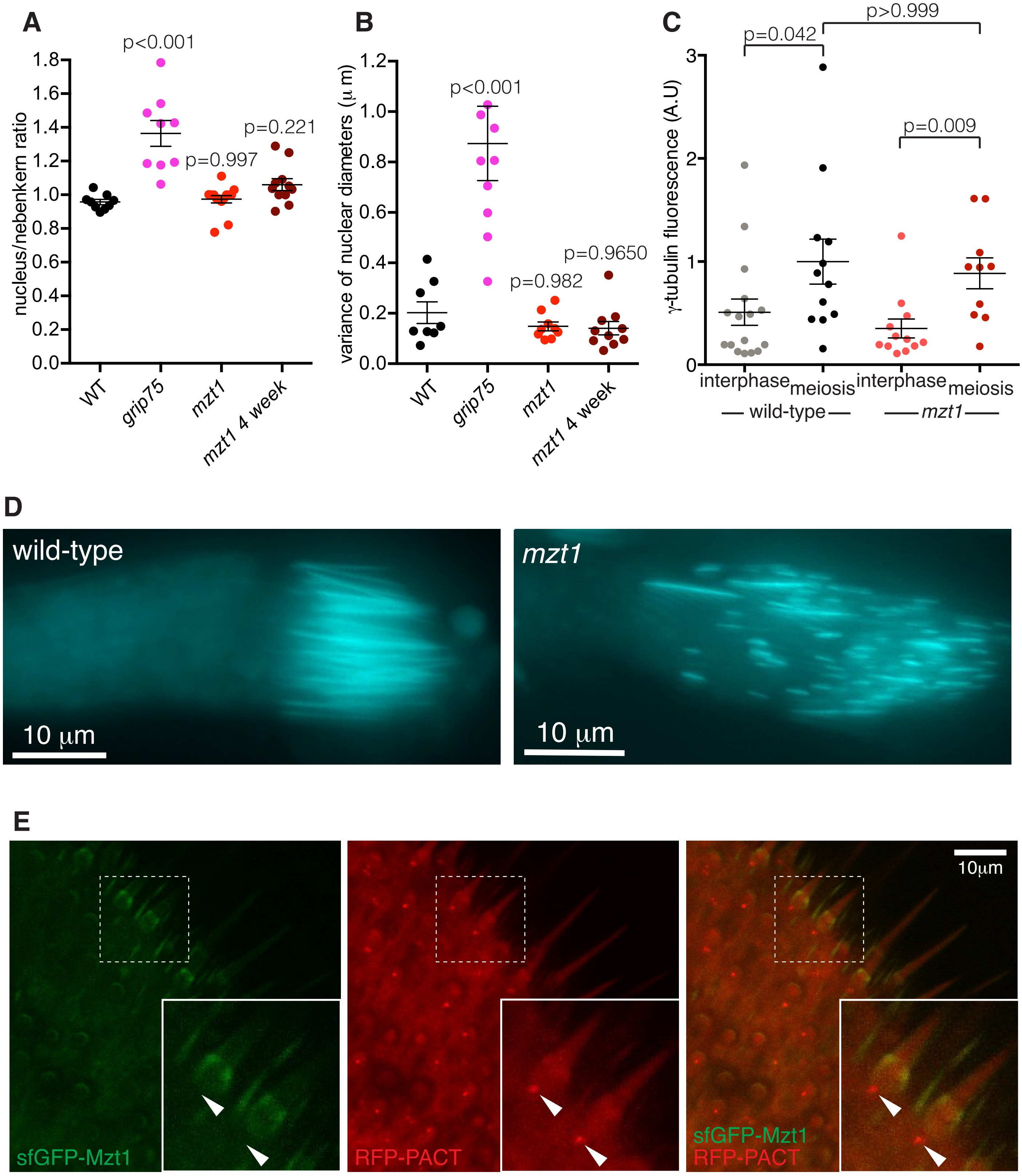
A
p<0.001
p=0.997
p=0.221
nucleus/nebenkern ratio
1.8
1.6
1.4
1.2
1.0
0.8
0.6
0.4
0.2
0.0
WT
grip75
mzt1
mzt1 4 week
B
p<0.001
p=0.982
p=0.9650
variance of nuclear diameters (μ m)
1.2
1.0
0.8
0.6
0.4
0.2
0.0
WT
grip75
mzt1
mzt1 4 week
C
p=0.042
p>0.999
p=0.009
γ-tubulin fluorescence (A.U)
3
2
1
0
interphase
meiosis
interphase
meiosis
wild-type
mzt1
D
wild-type
10 μm
mzt1
10 μm
E
sfGFP-Mzt1
RFP-PACT
10μm
sfGFP-Mzt1
RFP-PACT

Figure S3. Mzt1 is required for proper nuclei positioning in spermatids, but not for male meiosis, and does not localise to the basal bodies of cilia in sensory neurons, related to Figure 3. (**A**,**B**) Graphs show the results of an analysis of onion stage round spermatid cysts from pupal wild-type, pupal *grip75* mutant, pupal *mzt1* mutant or 4-week-old *mzt1* mutant flies, as indicated. Each data-point represents an average value from a cyst of cells. *mzt1* mutant cysts have a ~1:1 nucleus:nebenkern ratio (A) and display little variation in nuclear size (B), indicating that meiosis proceeds normally. (**C**) Graph shows the quantification of centrosomal γ-tubulin fluorescence in interphase and meiotic wild-type and *mzt1* mutants, as indicated; each data-point represents an average value from a cyst of cells. Note that the centrosomes in *mzt1* mutant meiotic spermatocytes can still recruit large amounts of γ-tubulin. (**D**) Images show spermatid bundles from live squashes of either wild-type (left) or *mzt1* mutant (right) testes incubated with Hoechst (DNA, blue). Autofluorescence reveals the position of the sperm tails. While the nuclei are all positioned at the tip of the bundle in the wild-type, they are more scattered in the *mzt1* mutant. Note that the Hoechst staining is not always evenly distributed along the length of an individual nucleus, such that the two ends can be brighter than the middle. (**E**) Antennae from flies co-expressing sfGFP-Mzt1 and RFP-PACT (a basal body marker) were dissected, fixed and mounted; images of the edge of the third antennal segment were taken on a confocal microscope. The insets in the bottom right of each image are enlargements of the boxed regions and the arrowheads point to basal bodies. Error bars represent SEM.

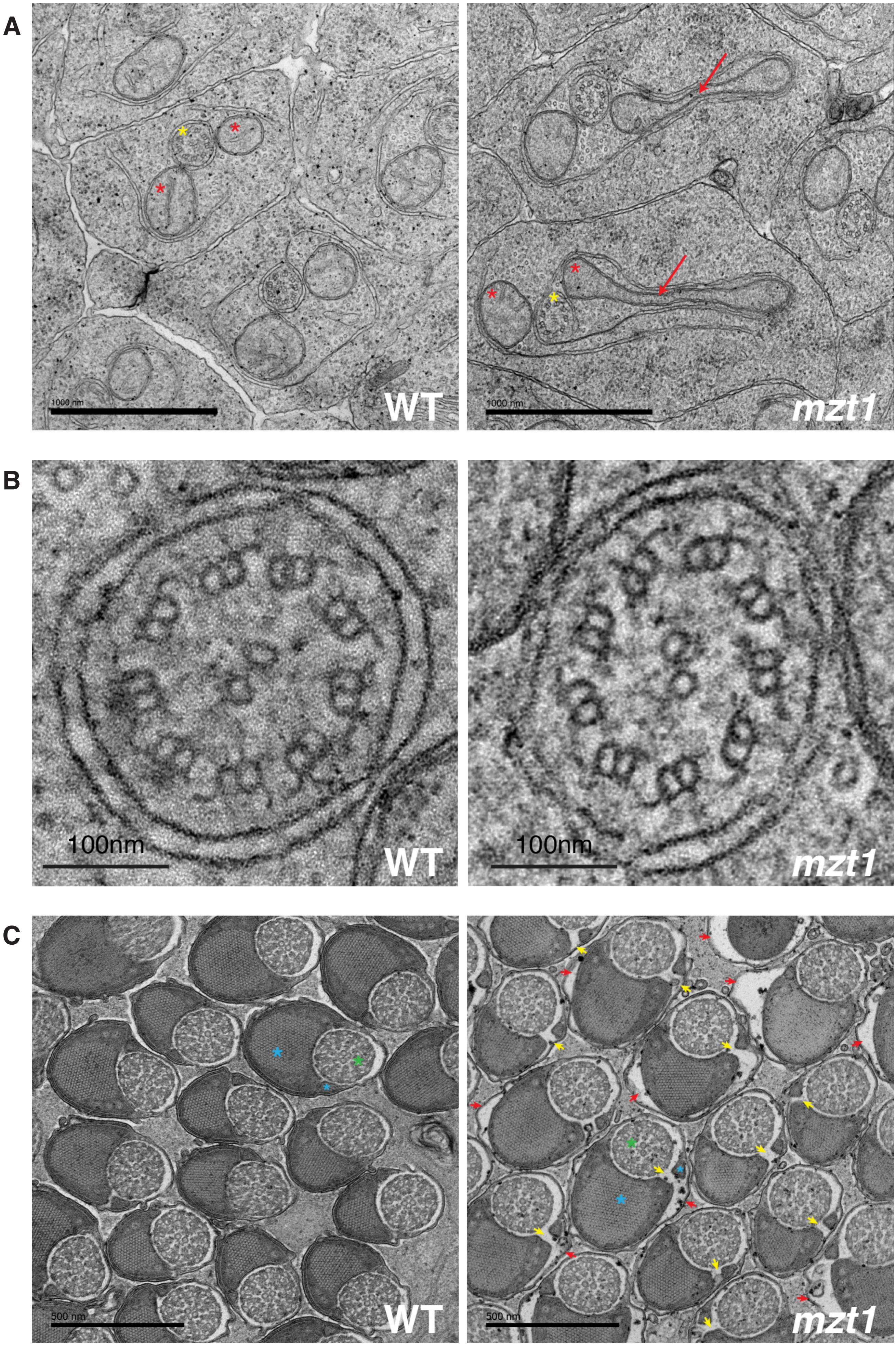
A
B
C
1000 nm
WT
1000 nm
mzt1
100nm
WT
100nm
mzt1
500 nm
WT
500 nm
mzt1

Figure S4. Transmission electron microscopy imaging of sperm tails reveals ultrastructural defects in *mzt1* mutants, related to Figure 3. (**A**) Images show cross sections of sperm tails from intermediate spermatids in either wild-type (left) or *mzt1* mutant (right) pupal testes. Axonemes and mitochondrial derivatives from an individual tail in each case are marked with a yellow asterisk and red asterisks, respectively. Misshapen, severely elongated mitochondrial derivatives in the mutant are indicated with red arrows. This type of severe elongation was seen in 10 out of 36 sperm tails examined in the mutant, but in only 1 out of 43 sperm tails in the wild-type. (**B**) Images show cross sections of axonemes from intermediate spermatids in either wild-type (left) or *mzt1* mutant (right) pupal testes. A 9+2 arrangement of microtubules can be seen in both cases. (**C**) Images show cross sections of individualised sperm tails from mature spermatids in either wild-type (left) or *mzt1* mutant (right) testes from 4-week-old flies. Axonemes and the major and minor mitochondrial derivatives from one of the tails in each case are marked with a green asterisk and blue asterisks, respectively. In the mutant, red arrowheads indicate where the membrane has separated from the major mitochondrial derivative (this occurred in 84.9% mutant tails, n=212, and in only 14.7% wild-type tails, n=68). Yellow arrowheads indicate unusually large gaps between the major and minor mitochondrial derivatives (this occurred in 71.7% mutant tails, n=212, and in only 4.4% wild-type tails, n=68).

Drosophila	Human
γ-tubulin37C (maternal)<br>γ-tubulin23C (ubiquitous)	γ-tubulin1 and γ-tubulin2
Grip84	GCP2
Grip91	GCP3
Grip75	GCP4
Grip128	GCP5
Grip163	GCP6
Grip71	NEDD1 / GCP-WD / GCP7
-	MOZART2A / GCP8A
-	MOZART2B / GCP8B
CG42787	MOZART1 / GCP9
nmdyn-D7	NME7 / NDK7
?	LGALS3BP

Table S1. Homologues of γ-TuRC proteins in *Drosophila* and human cells, related to Figures 1 and 3.
